# Supplementary material for: Computational Inference, Validation, and Analysis of 5’UTR-Leader Sequences of Alleles of Immunoglobulin Heavy Chain Variable Genes
Source: Front Immunol. 2021 Oct 4;12:730105. doi: 10.3389/fimmu.2021.730105 (PMC8521166; doi:10.3389/fimmu.2021.730105)
Supplement: Supplementary file 1 [file DataSheet_1.zip › Supplementary information/Supplementary_Figure_1.pdf]

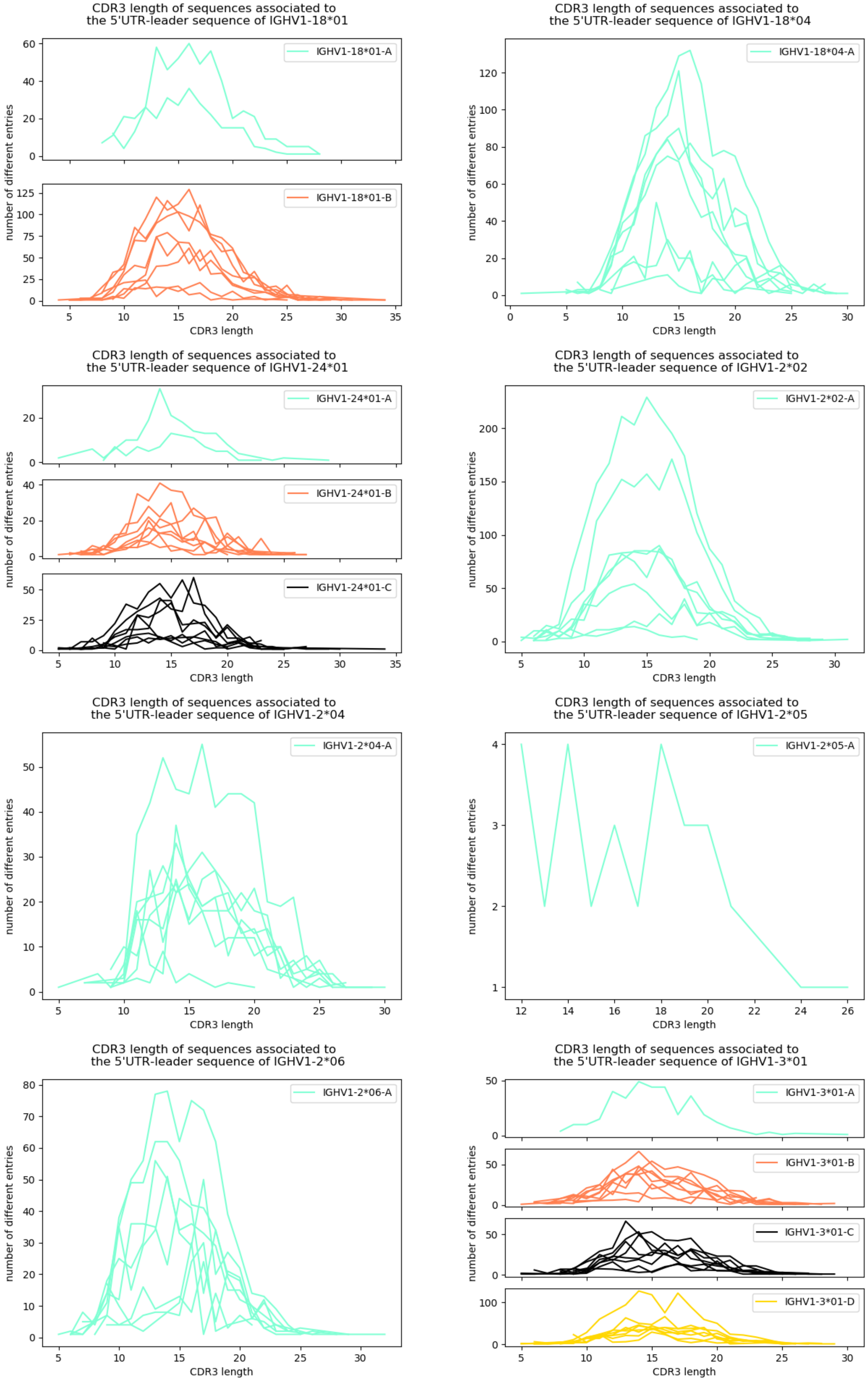
CDR3 length of sequences associated to the 5'UTR-leader sequence of IGHV1-18*01
IGHV1-18*01-A
IGHV1-18*01-B
CDR3 length of sequences associated to the 5'UTR-leader sequence of IGHV1-18*04
IGHV1-18*04-A
CDR3 length of sequences associated to the 5'UTR-leader sequence of IGHV1-24*01
IGHV1-24*01-A
IGHV1-24*01-B
IGHV1-24*01-C
CDR3 length of sequences associated to the 5'UTR-leader sequence of IGHV1-2*02
IGHV1-2*02-A
CDR3 length of sequences associated to the 5'UTR-leader sequence of IGHV1-2*04
IGHV1-2*04-A
CDR3 length of sequences associated to the 5'UTR-leader sequence of IGHV1-2*05
IGHV1-2*05-A
CDR3 length of sequences associated to the 5'UTR-leader sequence of IGHV1-2*06
IGHV1-2*06-A
CDR3 length of sequences associated to the 5'UTR-leader sequence of IGHV1-3*01
IGHV1-3*01-A
IGHV1-3*01-B
IGHV1-3*01-C
IGHV1-3*01-D
number of different entries
CDR3 length

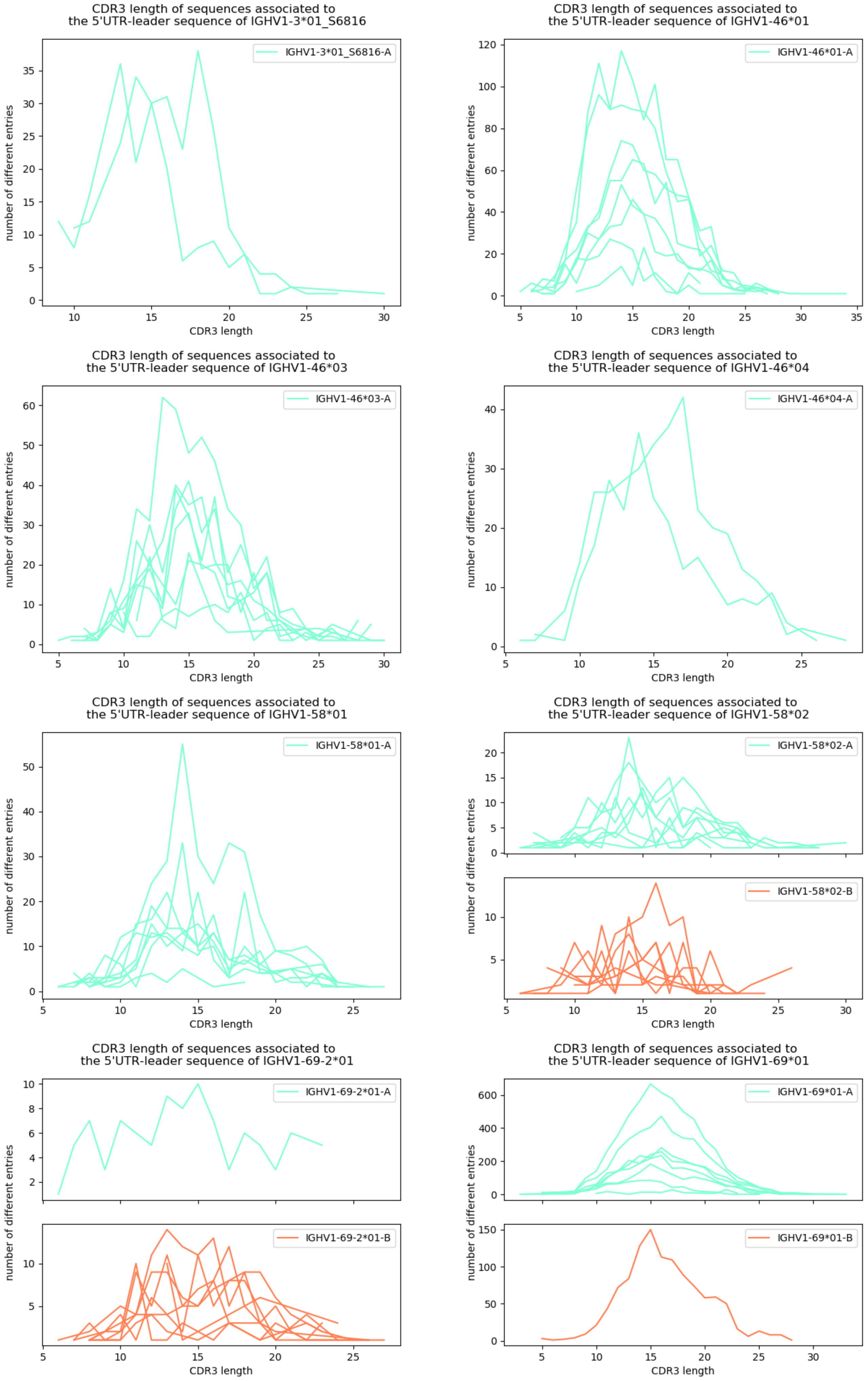
CDR3 length of sequences associated to
the 5'UTR-leader sequence of IGHV1-3*01_S6816
IGHV1-3*01_S6816-A
number of different entries
CDR3 length
CDR3 length of sequences associated to
the 5'UTR-leader sequence of IGHV1-46*01
IGHV1-46*01-A
number of different entries
CDR3 length
CDR3 length of sequences associated to
the 5'UTR-leader sequence of IGHV1-46*03
IGHV1-46*03-A
number of different entries
CDR3 length
CDR3 length of sequences associated to
the 5'UTR-leader sequence of IGHV1-46*04
IGHV1-46*04-A
number of different entries
CDR3 length
CDR3 length of sequences associated to
the 5'UTR-leader sequence of IGHV1-58*01
IGHV1-58*01-A
number of different entries
CDR3 length
CDR3 length of sequences associated to
the 5'UTR-leader sequence of IGHV1-58*02
IGHV1-58*02-A
IGHV1-58*02-B
number of different entries
CDR3 length
CDR3 length of sequences associated to
the 5'UTR-leader sequence of IGHV1-69-2*01
IGHV1-69-2*01-A
IGHV1-69-2*01-B
number of different entries
CDR3 length
CDR3 length of sequences associated to
the 5'UTR-leader sequence of IGHV1-69*01
IGHV1-69*01-A
IGHV1-69*01-B
number of different entries
CDR3 length

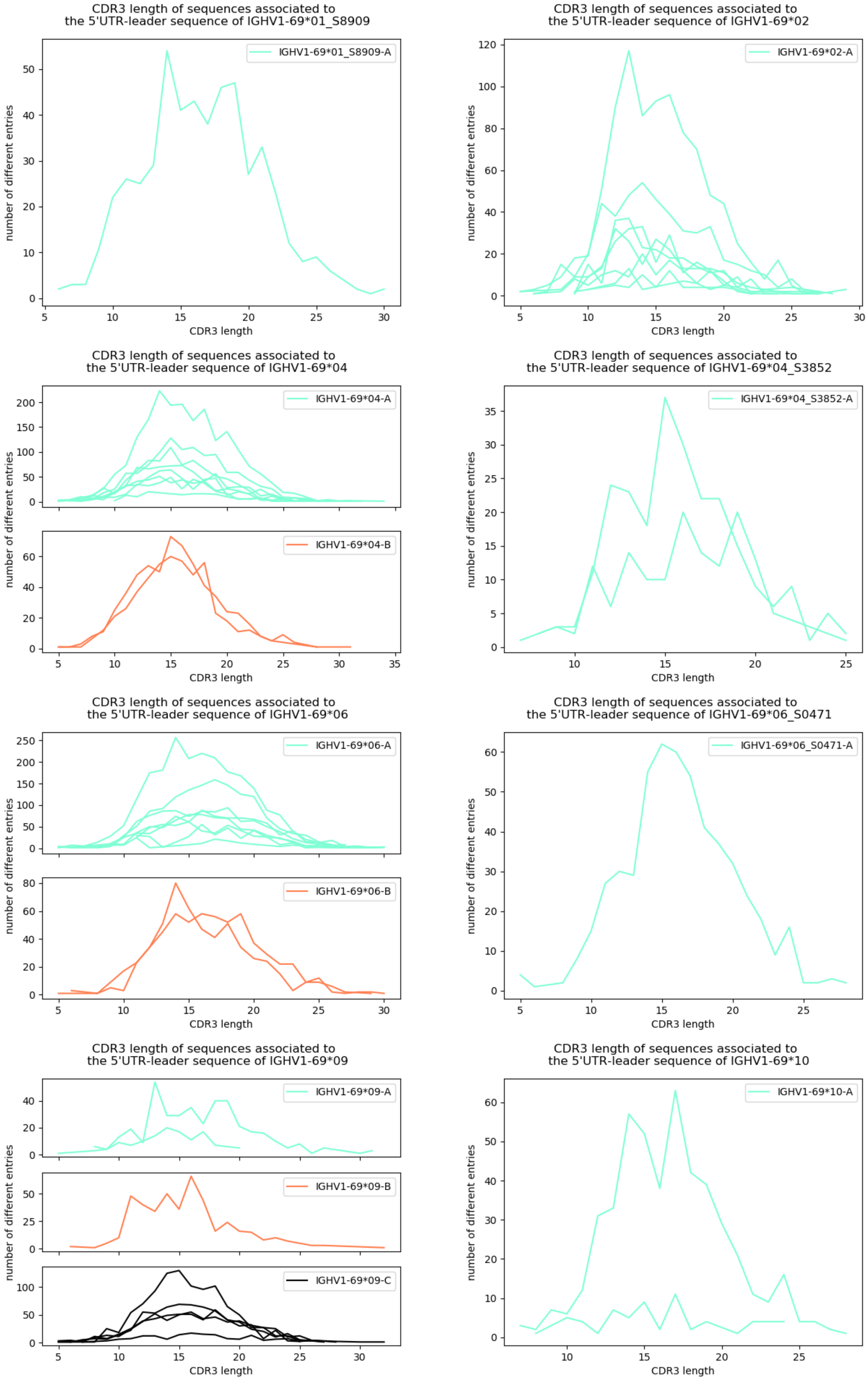
CDR3 length of sequences associated to the 5'UTR-leader sequence of IGHV1-69*01_S8909
IGHV1-69*01_S8909-A
CDR3 length of sequences associated to the 5'UTR-leader sequence of IGHV1-69*02
IGHV1-69*02-A
CDR3 length of sequences associated to the 5'UTR-leader sequence of IGHV1-69*04
IGHV1-69*04-A
IGHV1-69*04-B
CDR3 length of sequences associated to the 5'UTR-leader sequence of IGHV1-69*04_S3852
IGHV1-69*04_S3852-A
CDR3 length of sequences associated to the 5'UTR-leader sequence of IGHV1-69*06
IGHV1-69*06-A
IGHV1-69*06-B
CDR3 length of sequences associated to the 5'UTR-leader sequence of IGHV1-69*06_S0471
IGHV1-69*06_S0471-A
CDR3 length of sequences associated to the 5'UTR-leader sequence of IGHV1-69*09
IGHV1-69*09-A
IGHV1-69*09-B
IGHV1-69*09-C
CDR3 length of sequences associated to the 5'UTR-leader sequence of IGHV1-69*10
IGHV1-69*10-A
number of different entries
CDR3 length

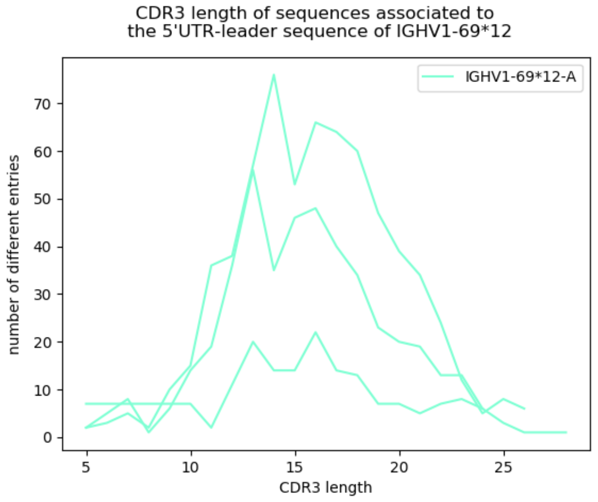
CDR3 length of sequences associated to
the 5'UTR-leader sequence of IGHV1-69*12
IGHV1-69*12-A
number of different entries
CDR3 length

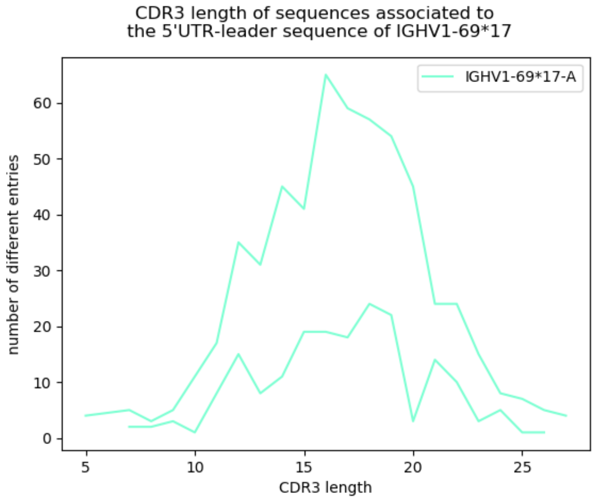
CDR3 length of sequences associated to
the 5'UTR-leader sequence of IGHV1-69*17
IGHV1-69*17-A
number of different entries
CDR3 length

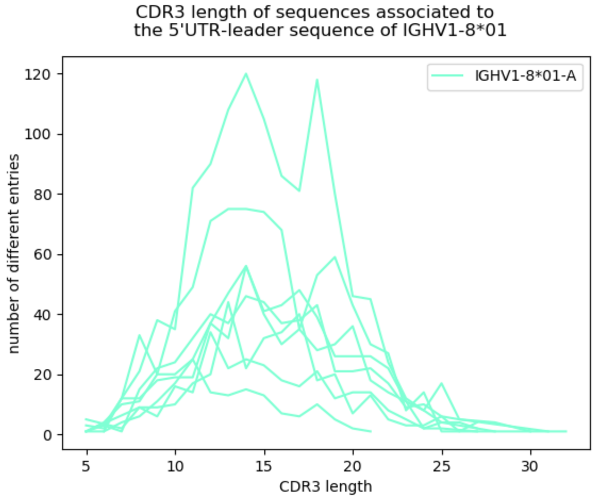
CDR3 length of sequences associated to
the 5'UTR-leader sequence of IGHV1-8*01
IGHV1-8*01-A
number of different entries
CDR3 length

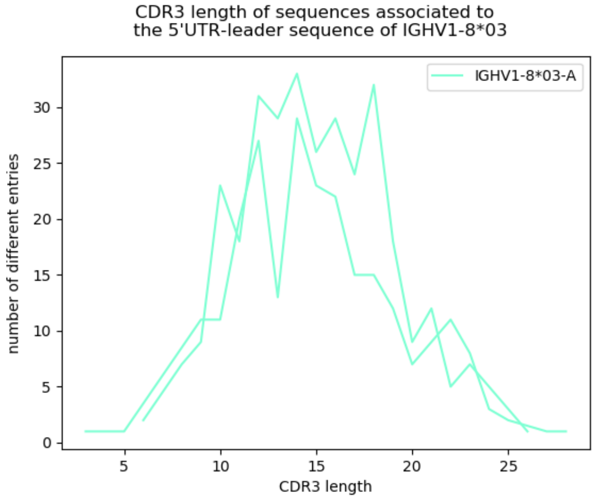
CDR3 length of sequences associated to
the 5'UTR-leader sequence of IGHV1-8*03
IGHV1-8*03-A
number of different entries
CDR3 length

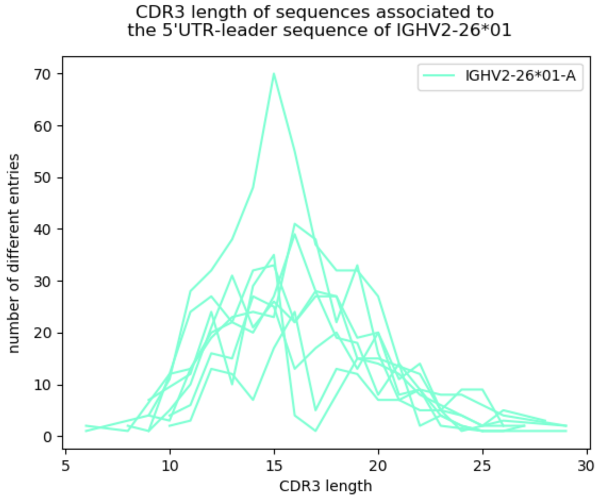
CDR3 length of sequences associated to
the 5'UTR-leader sequence of IGHV2-26*01
IGHV2-26*01-A
number of different entries
CDR3 length

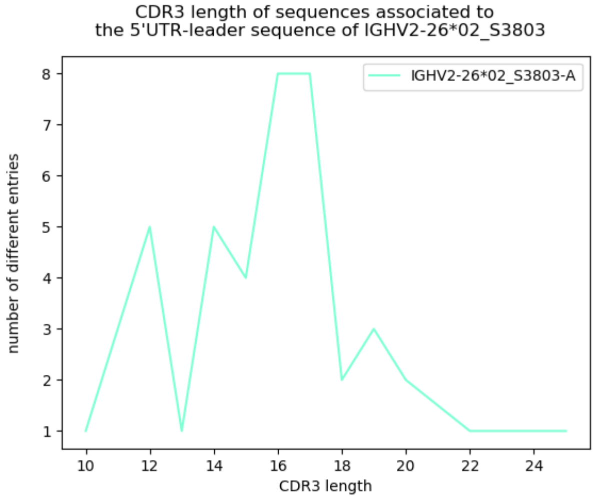
CDR3 length of sequences associated to
the 5'UTR-leader sequence of IGHV2-26*02_S3803
IGHV2-26*02_S3803-A
number of different entries
CDR3 length

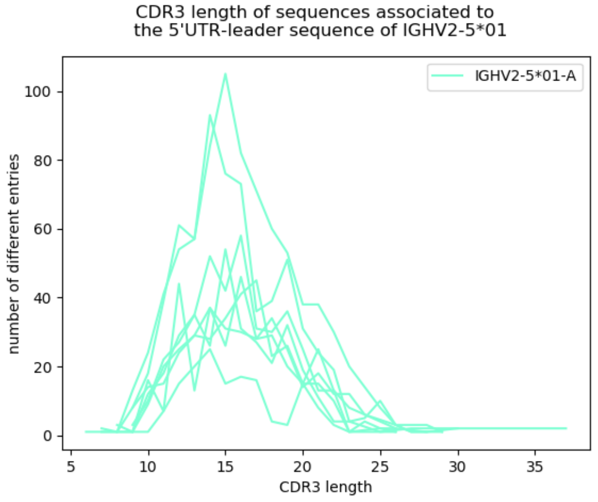
CDR3 length of sequences associated to
the 5'UTR-leader sequence of IGHV2-5*01
IGHV2-5*01-A
number of different entries
CDR3 length

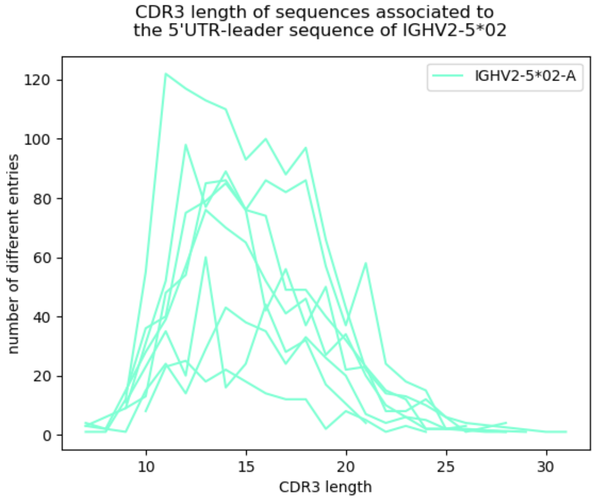
CDR3 length of sequences associated to
the 5'UTR-leader sequence of IGHV2-5*02
IGHV2-5*02-A
number of different entries
CDR3 length

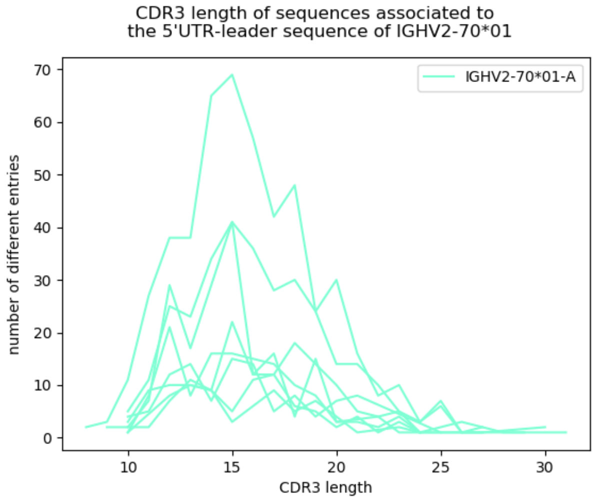
CDR3 length of sequences associated to
the 5'UTR-leader sequence of IGHV2-70*01
IGHV2-70*01-A
number of different entries
CDR3 length

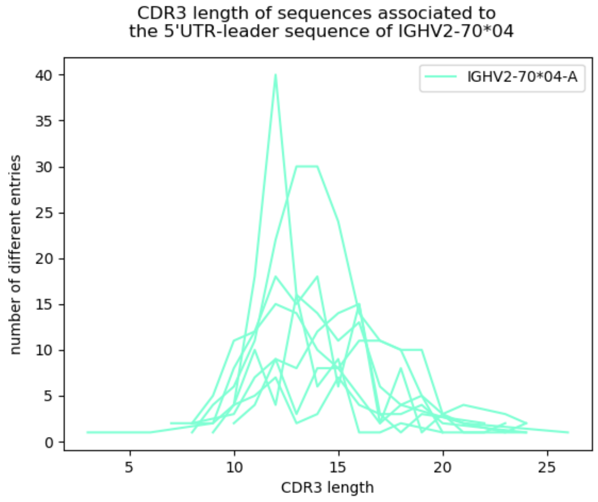
CDR3 length of sequences associated to
the 5'UTR-leader sequence of IGHV2-70*04
IGHV2-70*04-A
number of different entries
CDR3 length

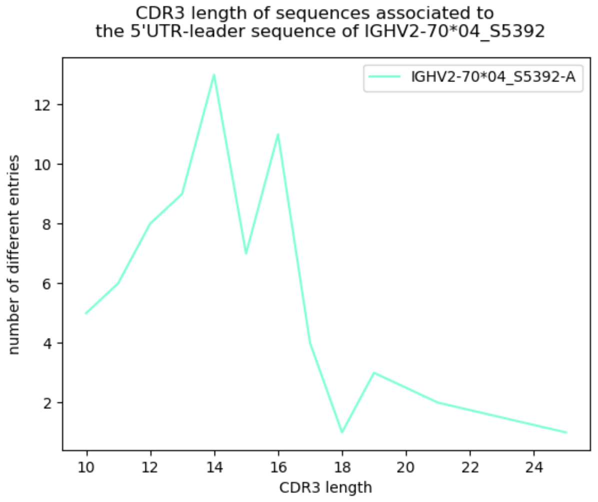
CDR3 length of sequences associated to
the 5'UTR-leader sequence of IGHV2-70*04_S5392
IGHV2-70*04_S5392-A
number of different entries
CDR3 length

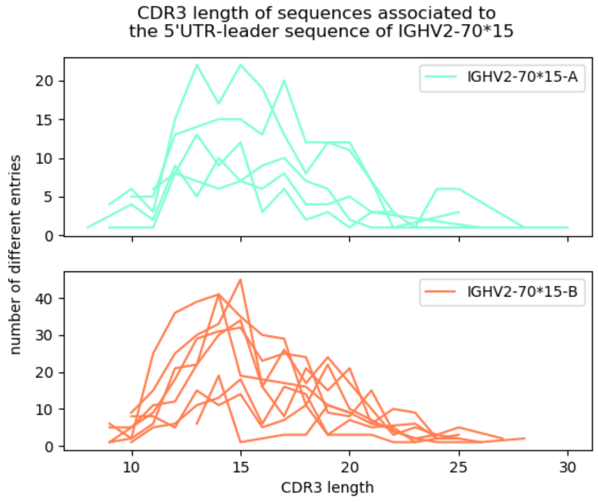
CDR3 length of sequences associated to
the 5'UTR-leader sequence of IGHV2-70*15
IGHV2-70*15-A
IGHV2-70*15-B
number of different entries
CDR3 length

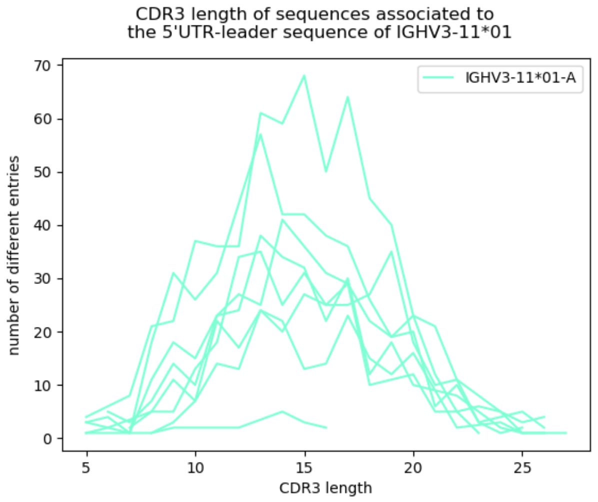
CDR3 length of sequences associated to
the 5'UTR-leader sequence of IGHV3-11*01
IGHV3-11*01-A
number of different entries
CDR3 length

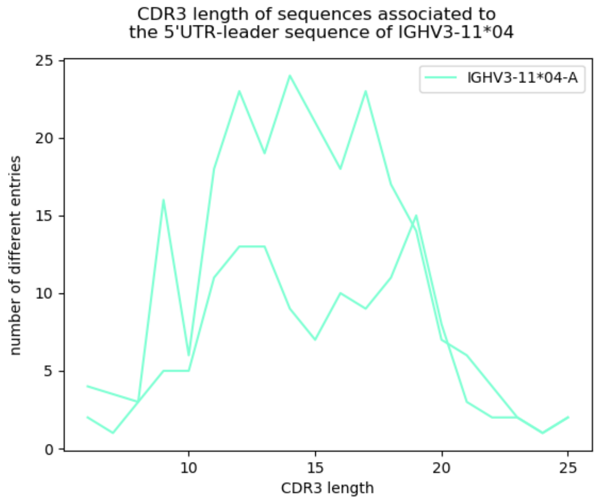
CDR3 length of sequences associated to
the 5'UTR-leader sequence of IGHV3-11*04
IGHV3-11*04-A
number of different entries
CDR3 length

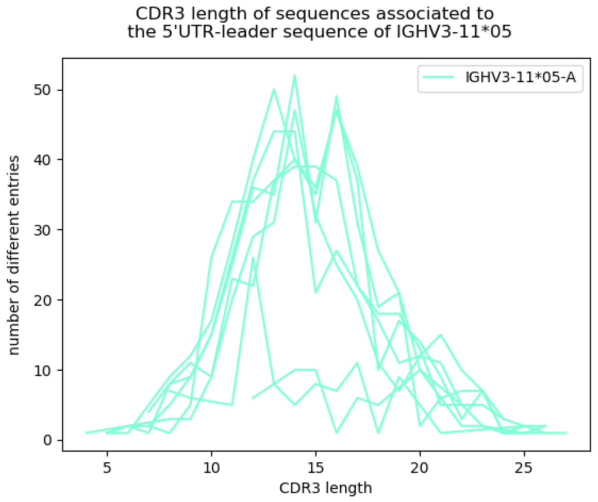
CDR3 length of sequences associated to
the 5'UTR-leader sequence of IGHV3-11*05
IGHV3-11*05-A
number of different entries
CDR3 length

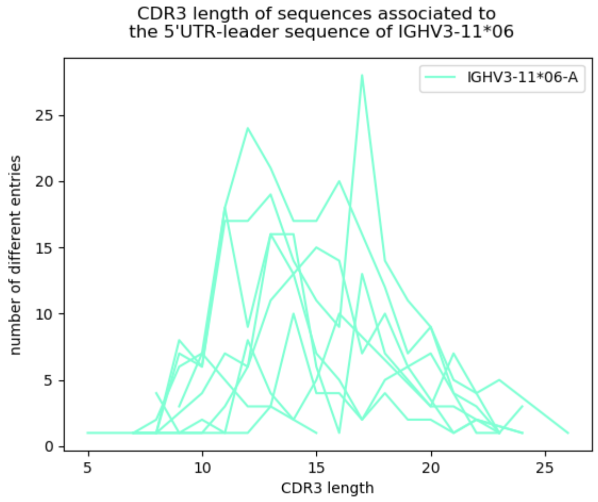
CDR3 length of sequences associated to
the 5'UTR-leader sequence of IGHV3-11*06
IGHV3-11*06-A
number of different entries
CDR3 length

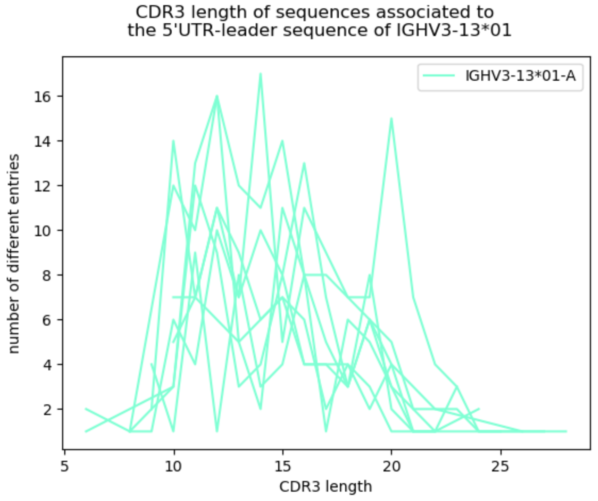
CDR3 length of sequences associated to
the 5'UTR-leader sequence of IGHV3-13*01
IGHV3-13*01-A
number of different entries
CDR3 length

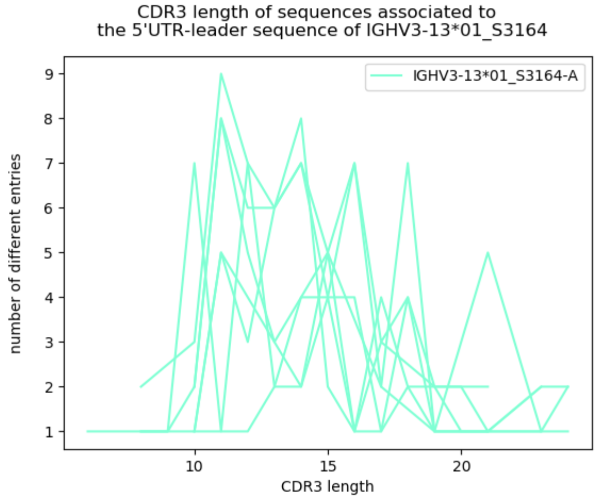
CDR3 length of sequences associated to
the 5'UTR-leader sequence of IGHV3-13*01_S3164
IGHV3-13*01_S3164-A
number of different entries
CDR3 length

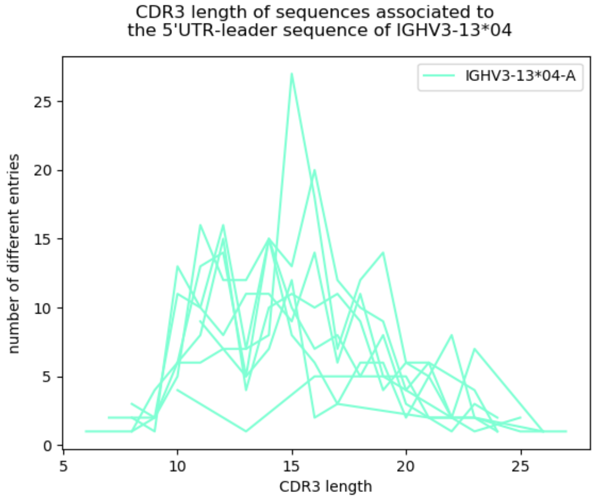
CDR3 length of sequences associated to
the 5'UTR-leader sequence of IGHV3-13*04
IGHV3-13*04-A
number of different entries
CDR3 length

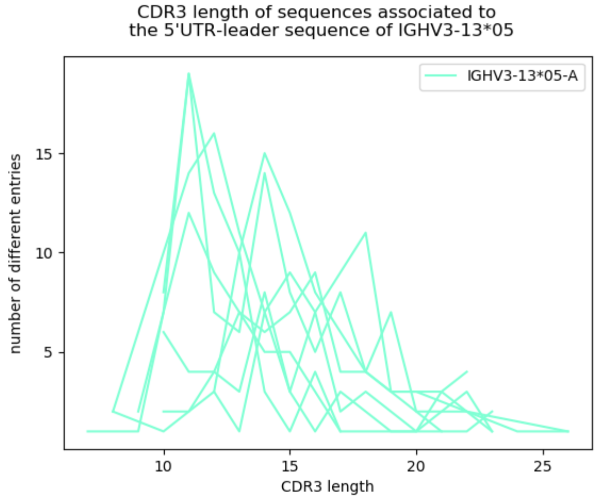
CDR3 length of sequences associated to
the 5'UTR-leader sequence of IGHV3-13*05
IGHV3-13*05-A
number of different entries
CDR3 length

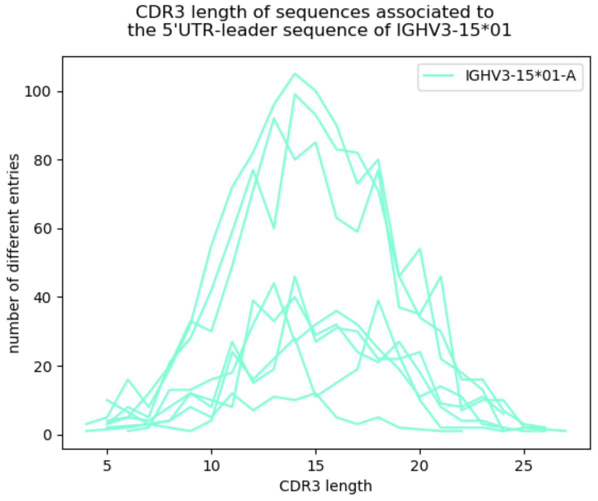
CDR3 length of sequences associated to
the 5'UTR-leader sequence of IGHV3-15*01
IGHV3-15*01-A
number of different entries
CDR3 length

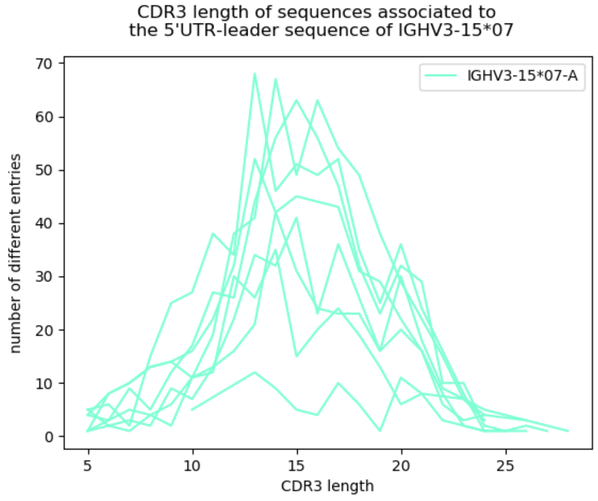
CDR3 length of sequences associated to
the 5'UTR-leader sequence of IGHV3-15*07
IGHV3-15*07-A
number of different entries
CDR3 length

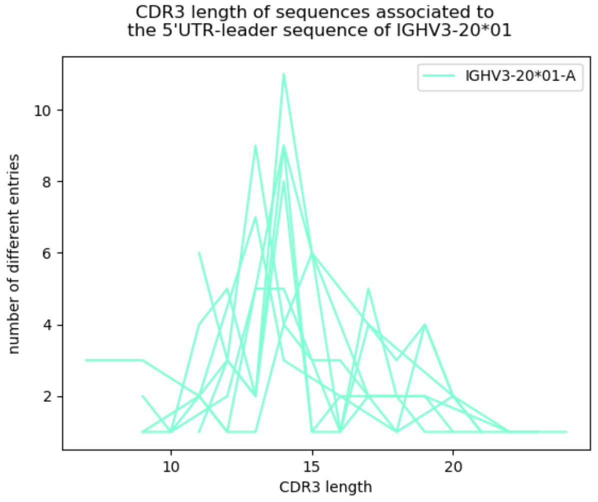
CDR3 length of sequences associated to
the 5'UTR-leader sequence of IGHV3-20*01
IGHV3-20*01-A
number of different entries
CDR3 length

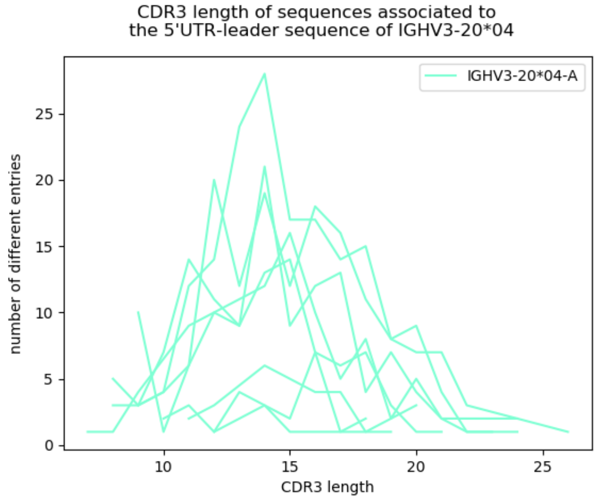
CDR3 length of sequences associated to
the 5'UTR-leader sequence of IGHV3-20*04
IGHV3-20*04-A
number of different entries
CDR3 length

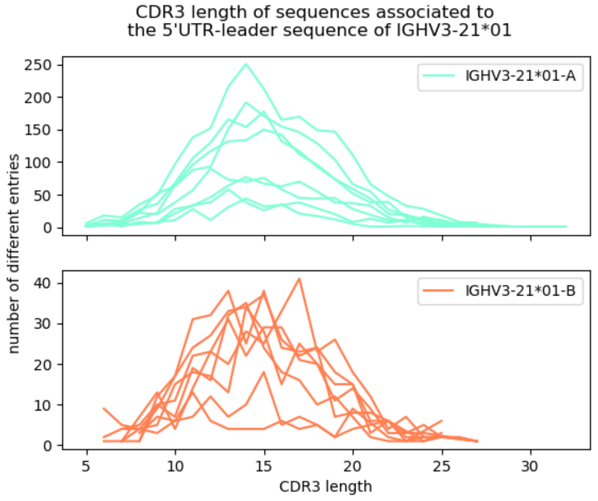
CDR3 length of sequences associated to
the 5'UTR-leader sequence of IGHV3-21*01
IGHV3-21*01-A
IGHV3-21*01-B
number of different entries
CDR3 length

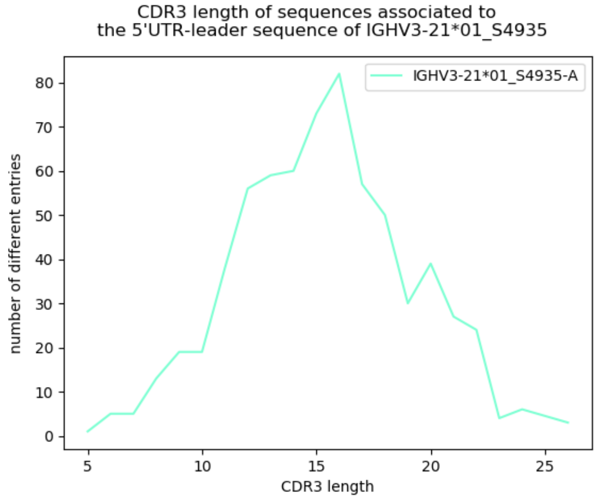
CDR3 length of sequences associated to
the 5'UTR-leader sequence of IGHV3-21*01_S4935
IGHV3-21*01_S4935-A
number of different entries
CDR3 length

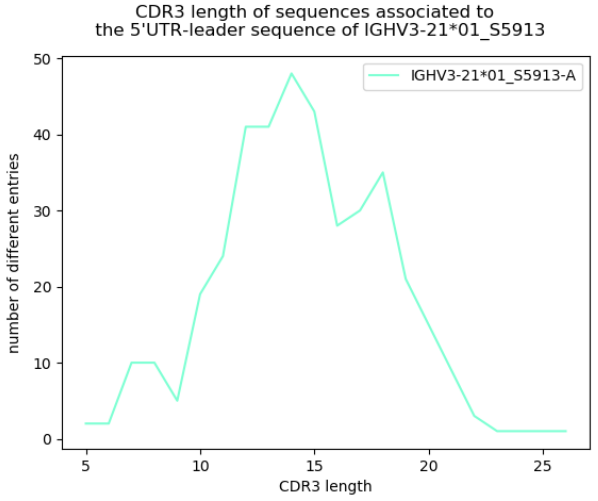
CDR3 length of sequences associated to
the 5'UTR-leader sequence of IGHV3-21*01_S5913
IGHV3-21*01_S5913-A
number of different entries
CDR3 length

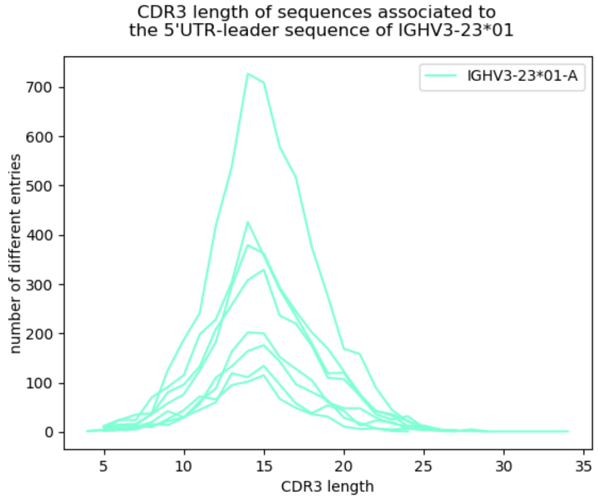
CDR3 length of sequences associated to
the 5'UTR-leader sequence of IGHV3-23*01
IGHV3-23*01-A
number of different entries
CDR3 length

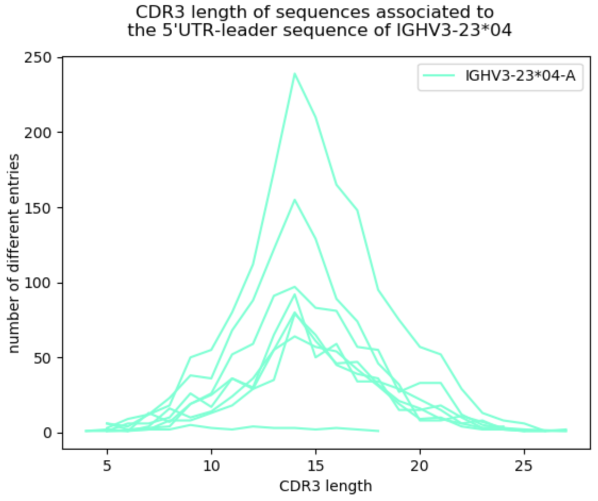
CDR3 length of sequences associated to
the 5'UTR-leader sequence of IGHV3-23*04
IGHV3-23*04-A
number of different entries
CDR3 length

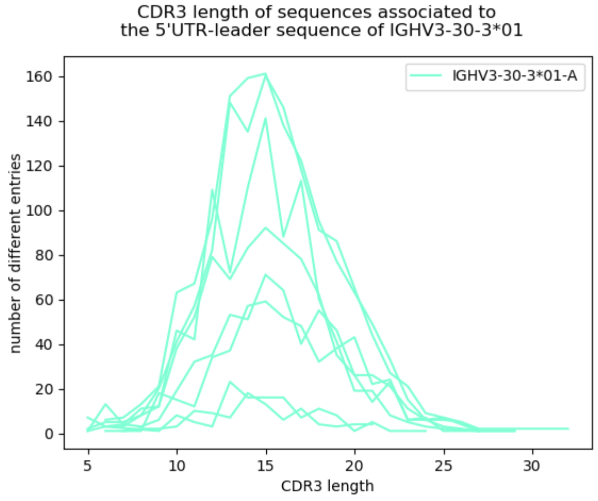
CDR3 length of sequences associated to
the 5'UTR-leader sequence of IGHV3-30-3*01
IGHV3-30-3*01-A
number of different entries
CDR3 length

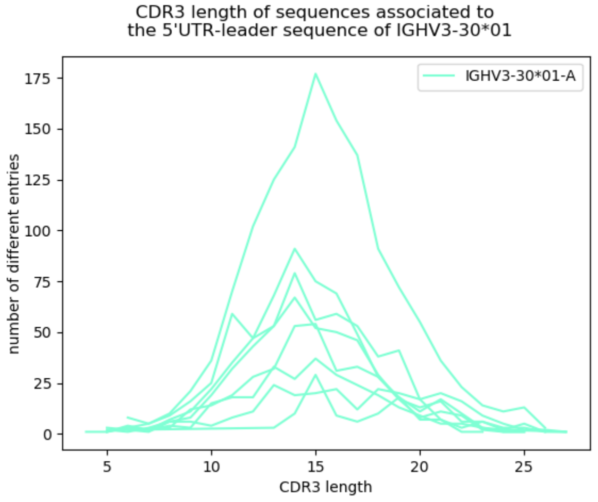
CDR3 length of sequences associated to
the 5'UTR-leader sequence of IGHV3-30*01
IGHV3-30*01-A
number of different entries
CDR3 length

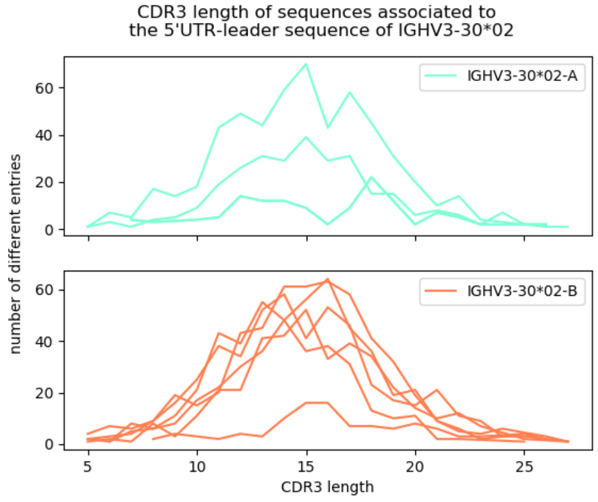
CDR3 length of sequences associated to
the 5'UTR-leader sequence of IGHV3-30*02
IGHV3-30*02-A
IGHV3-30*02-B
number of different entries
CDR3 length

CDR3 length of sequences associated to
the 5'UTR-leader sequence of IGHV3-30*02_S4989

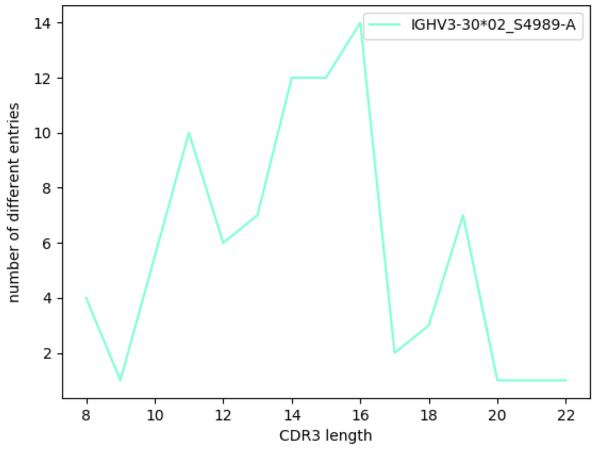

CDR3 length of sequences associated to
the 5'UTR-leader sequence of IGHV3-30*03

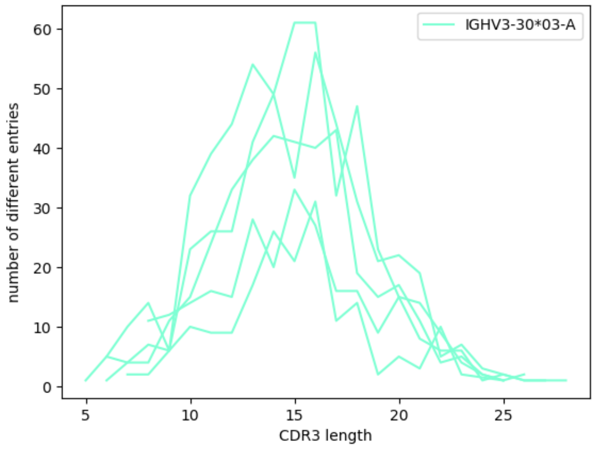

CDR3 length of sequences associated to
the 5'UTR-leader sequence of IGHV3-30*04

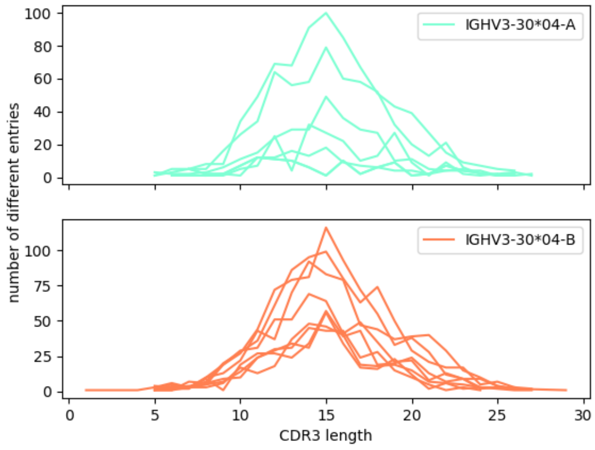

CDR3 length of sequences associated to
the 5'UTR-leader sequence of IGHV3-30*04_S7005

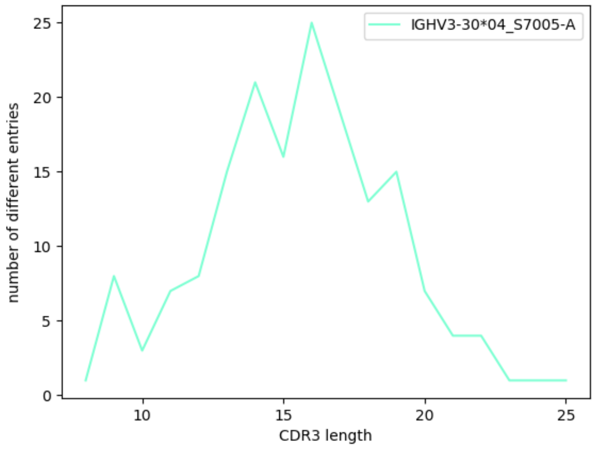

CDR3 length of sequences associated to
the 5'UTR-leader sequence of IGHV3-30*18

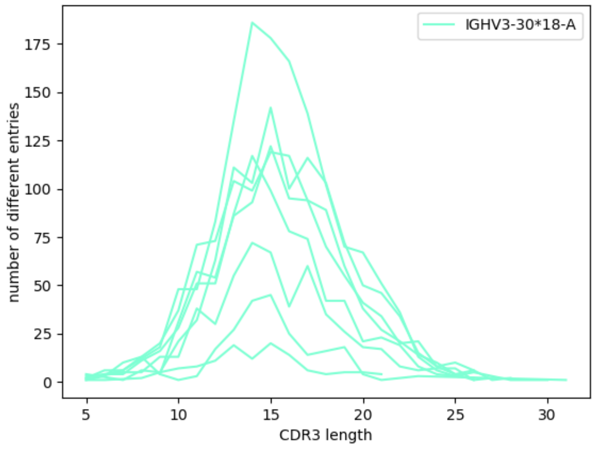

CDR3 length of sequences associated to
the 5'UTR-leader sequence of IGHV3-30*19_S5956

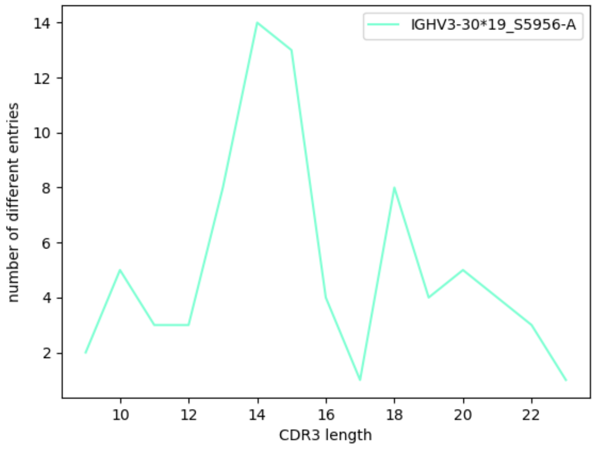

CDR3 length of sequences associated to
the 5'UTR-leader sequence of IGHV3-33*01

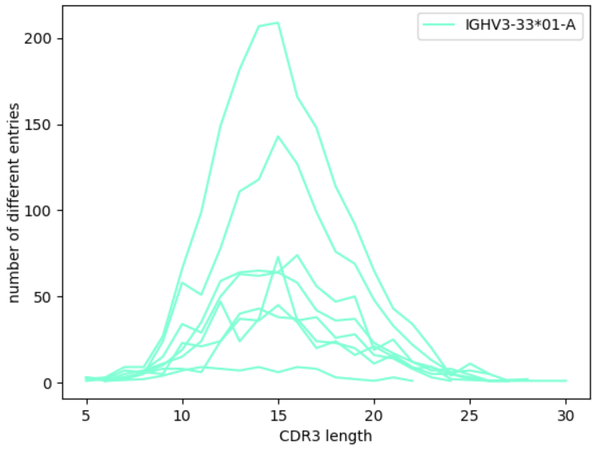

CDR3 length of sequences associated to
the 5'UTR-leader sequence of IGHV3-33*01_S3418

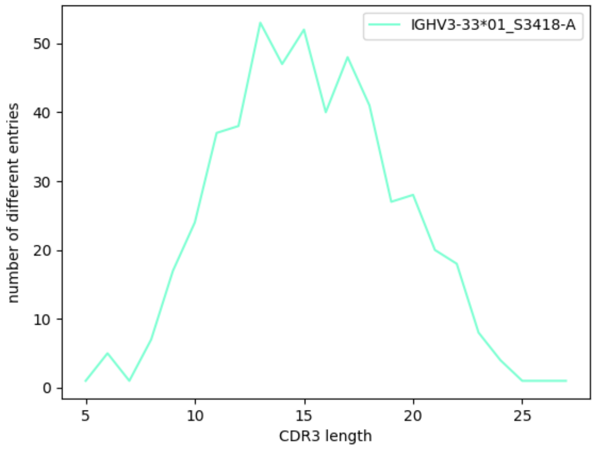

CDR3 length of sequences associated to
the 5'UTR-leader sequence of IGHV3-43D*03

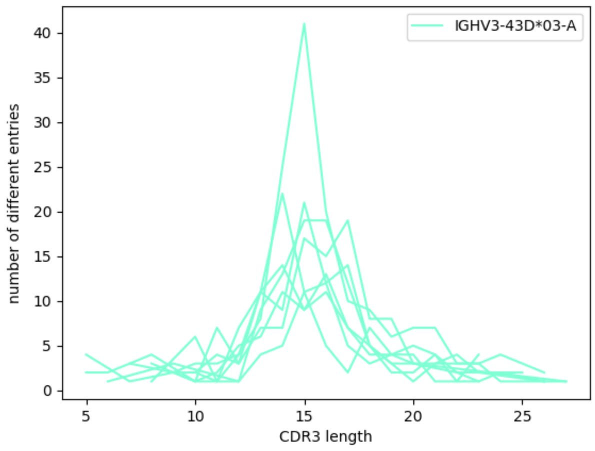

CDR3 length of sequences associated to
the 5'UTR-leader sequence of IGHV3-43D*04

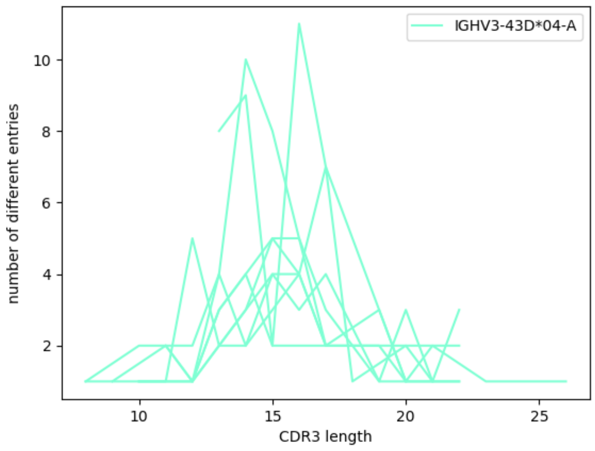

CDR3 length of sequences associated to
the 5'UTR-leader sequence of IGHV3-43D*04_S5432

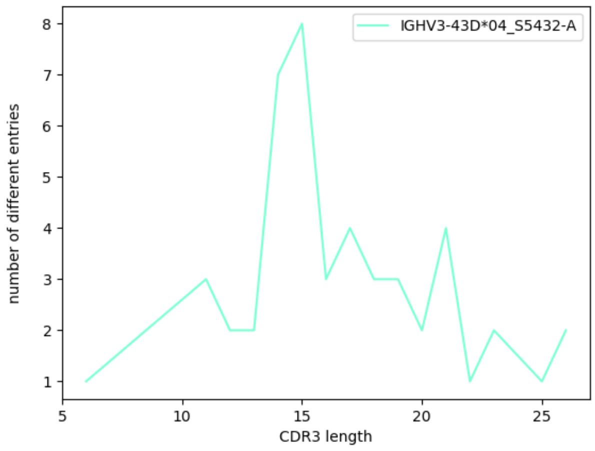

CDR3 length of sequences associated to
the 5'UTR-leader sequence of IGHV3-43*01

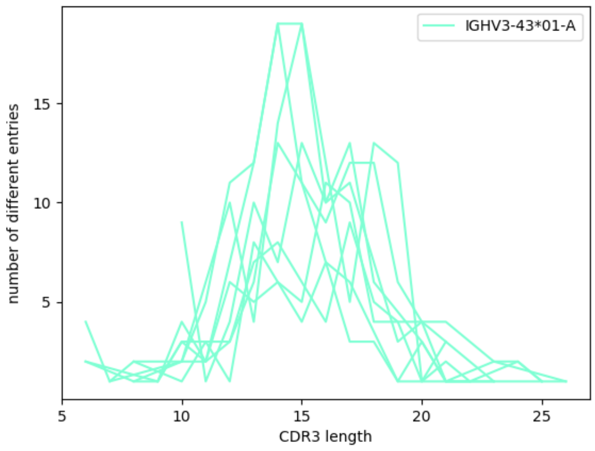

CDR3 length of sequences associated to
the 5'UTR-leader sequence of IGHV3-43*02

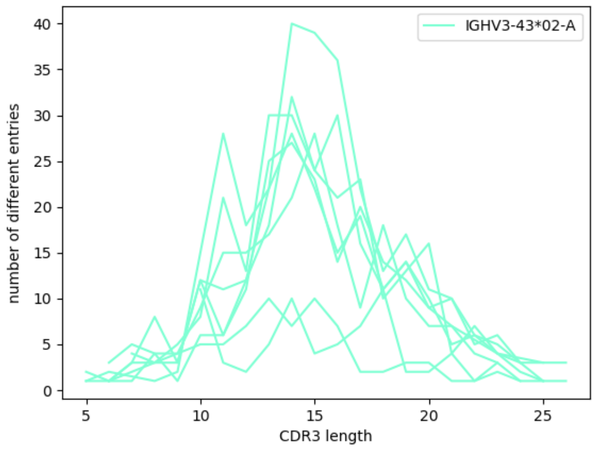

CDR3 length of sequences associated to
the 5'UTR-leader sequence of IGHV3-48*01

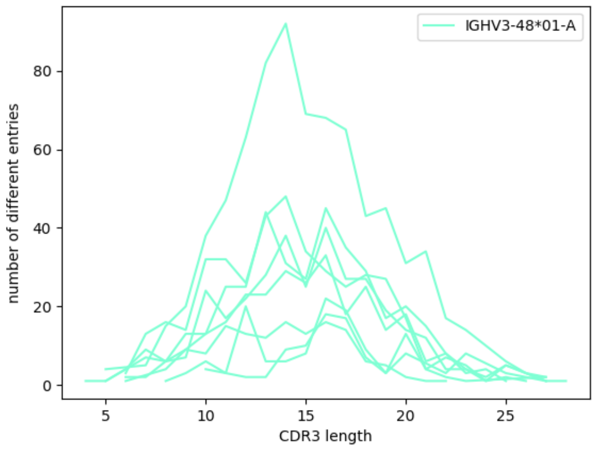

CDR3 length of sequences associated to
the 5'UTR-leader sequence of IGHV3-48*02

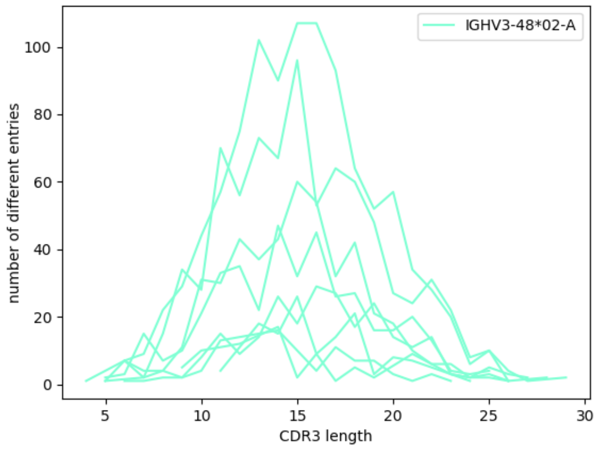

CDR3 length of sequences associated to
the 5'UTR-leader sequence of IGHV3-48*03

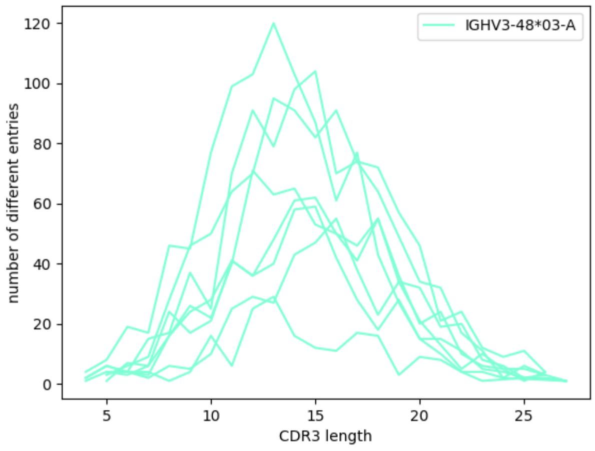

CDR3 length of sequences associated to
the 5'UTR-leader sequence of IGHV3-48*04

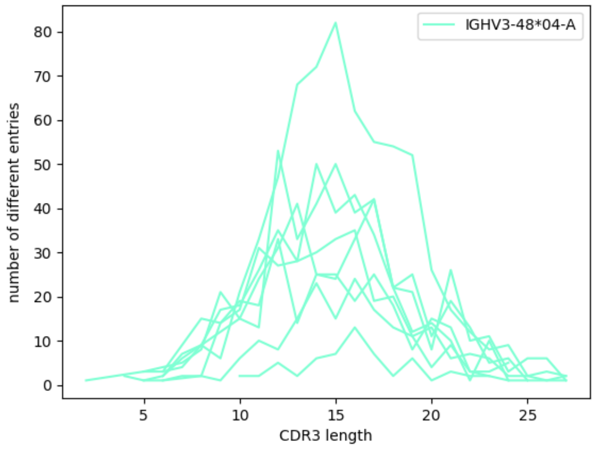

CDR3 length of sequences associated to
the 5'UTR-leader sequence of IGHV3-49*03

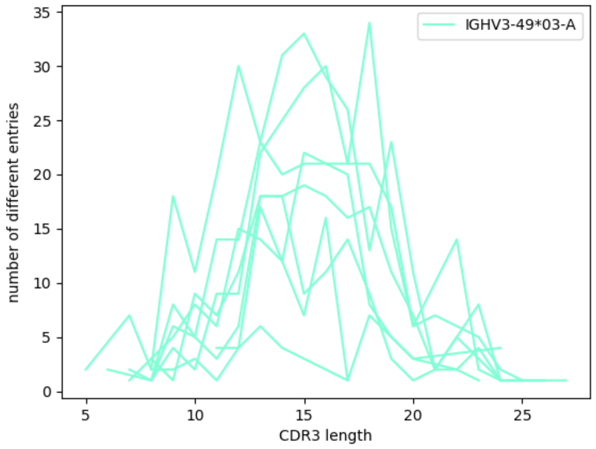

CDR3 length of sequences associated to
the 5'UTR-leader sequence of IGHV3-49*04

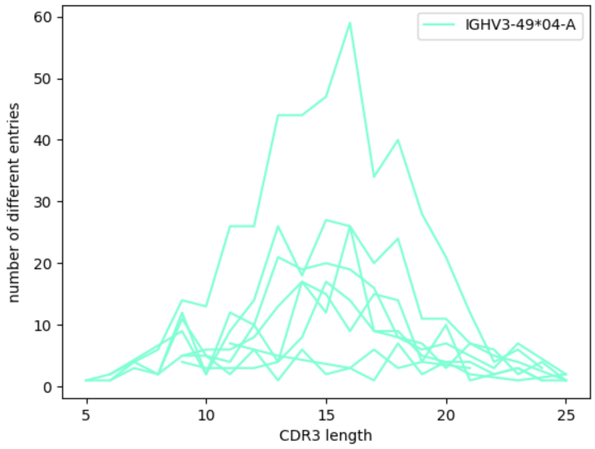

CDR3 length of sequences associated to
the 5'UTR-leader sequence of IGHV3-49*05

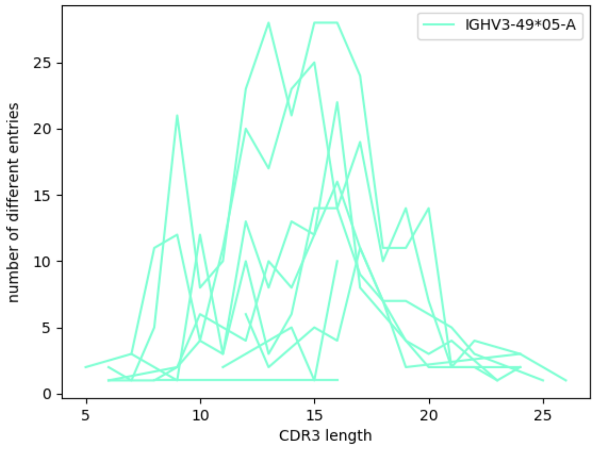

CDR3 length of sequences associated to
the 5'UTR-leader sequence of IGHV3-53*01

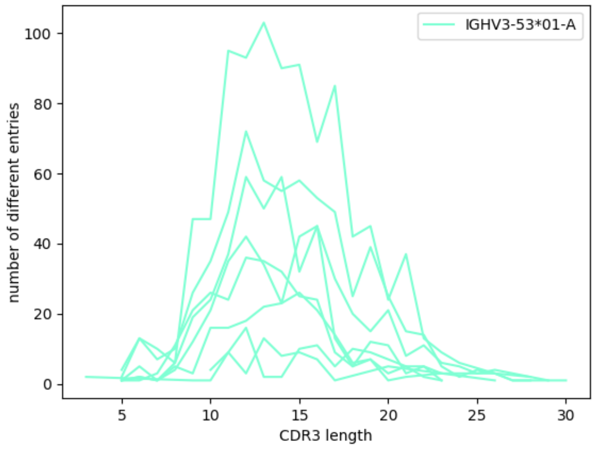

CDR3 length of sequences associated to
the 5'UTR-leader sequence of IGHV3-53*02

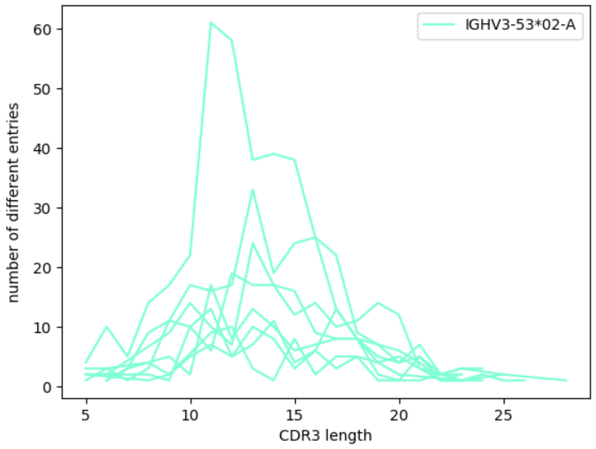

CDR3 length of sequences associated to
the 5'UTR-leader sequence of IGHV3-53*02_S9017

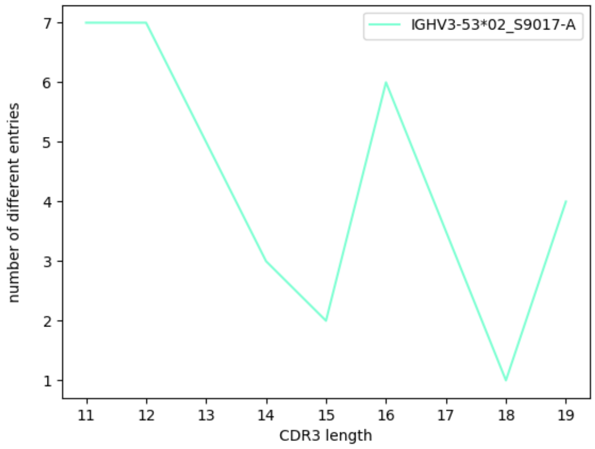

CDR3 length of sequences associated to
the 5'UTR-leader sequence of IGHV3-53*04

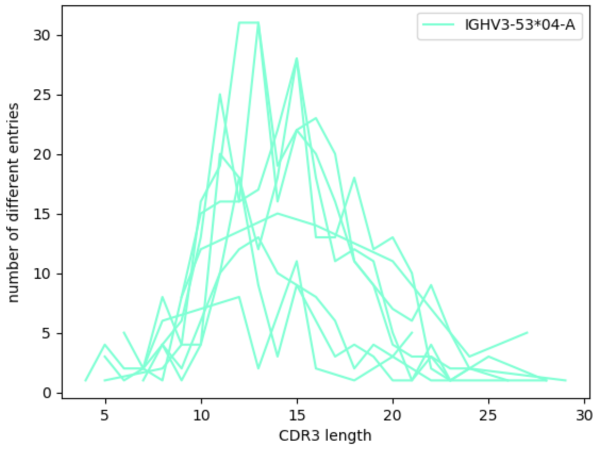

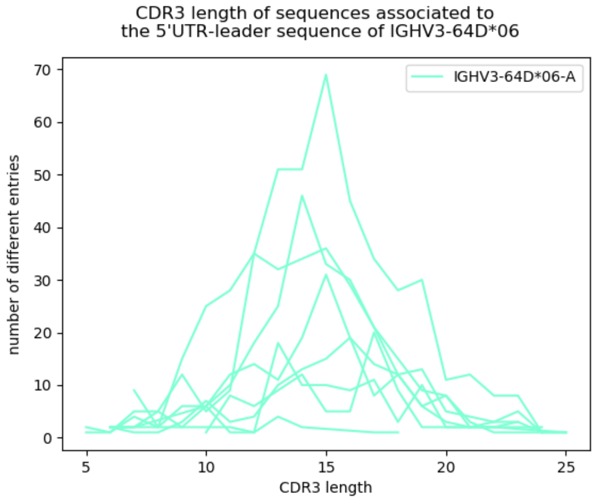
CDR3 length of sequences associated to
the 5'UTR-leader sequence of IGHV3-64D*06
IGHV3-64D*06-A
number of different entries
CDR3 length

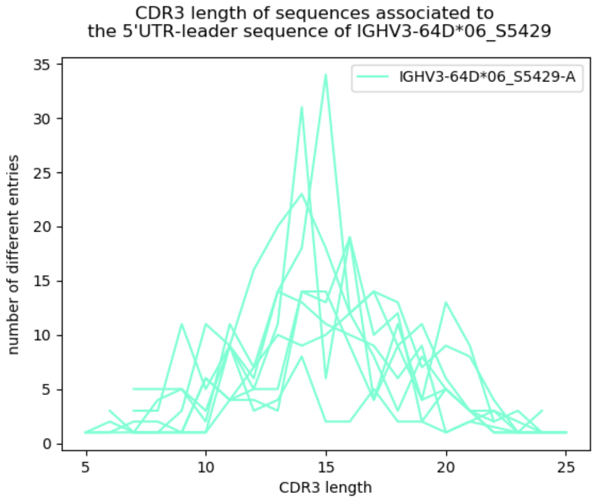
CDR3 length of sequences associated to
the 5'UTR-leader sequence of IGHV3-64D*06_S5429
IGHV3-64D*06_S5429-A
number of different entries
CDR3 length

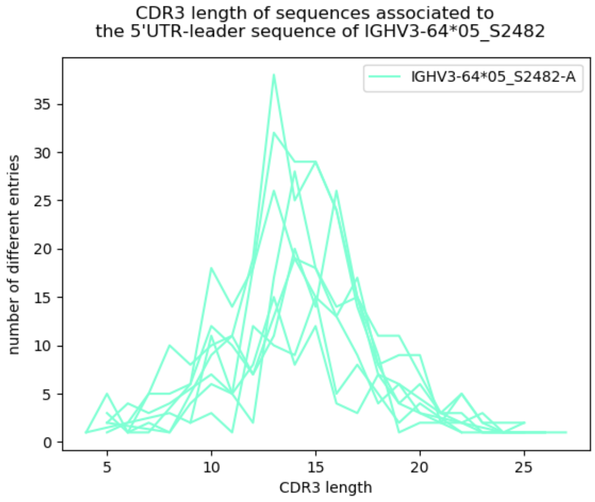
CDR3 length of sequences associated to
the 5'UTR-leader sequence of IGHV3-64*05_S2482
IGHV3-64*05_S2482-A
number of different entries
CDR3 length

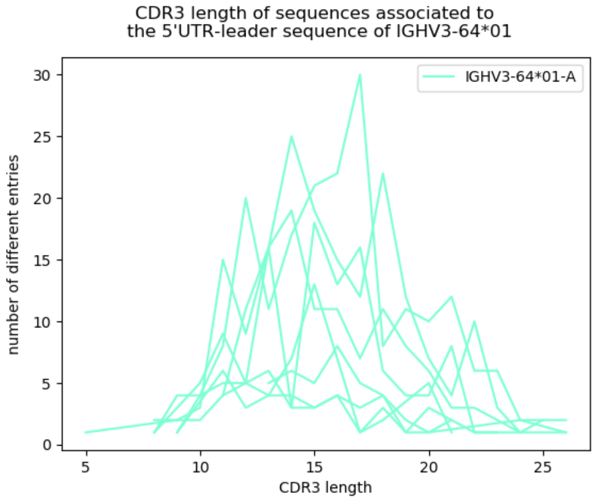
CDR3 length of sequences associated to
the 5'UTR-leader sequence of IGHV3-64*01
IGHV3-64*01-A
number of different entries
CDR3 length

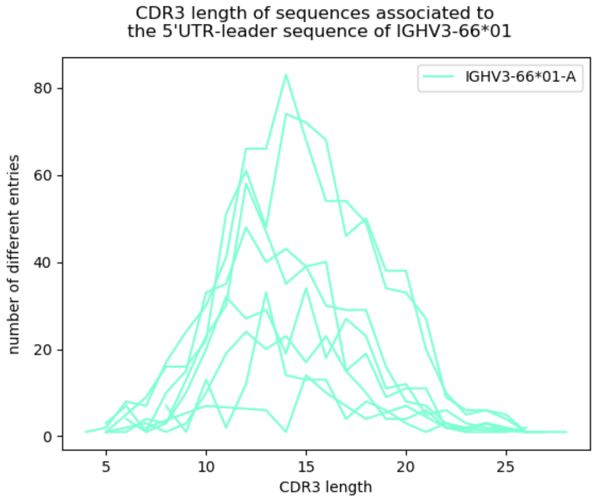
CDR3 length of sequences associated to
the 5'UTR-leader sequence of IGHV3-66*01
IGHV3-66*01-A
number of different entries
CDR3 length

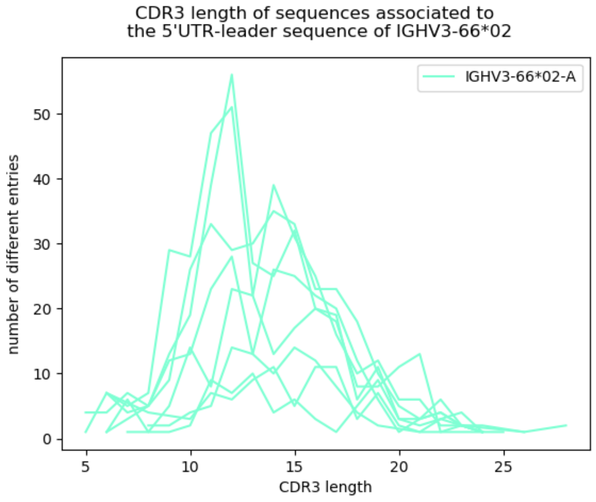
CDR3 length of sequences associated to
the 5'UTR-leader sequence of IGHV3-66*02
IGHV3-66*02-A
number of different entries
CDR3 length

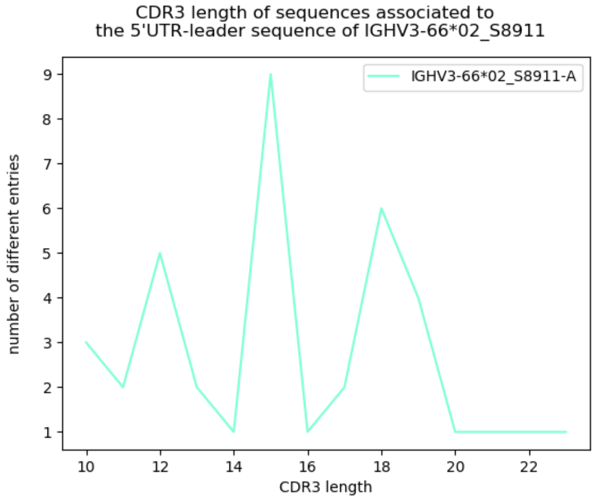
CDR3 length of sequences associated to
the 5'UTR-leader sequence of IGHV3-66*02_S8911
IGHV3-66*02_S8911-A
number of different entries
CDR3 length

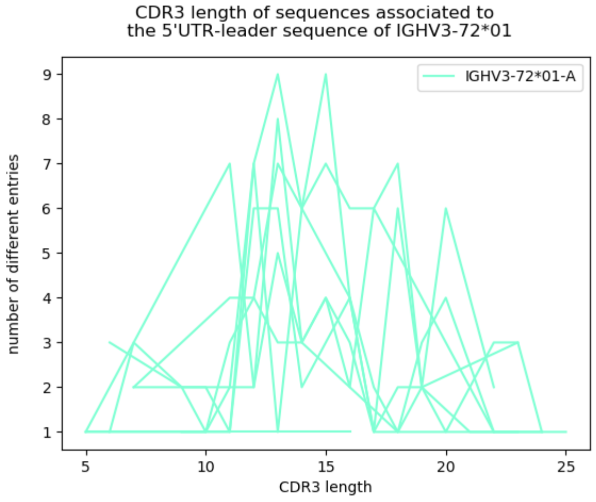
CDR3 length of sequences associated to
the 5'UTR-leader sequence of IGHV3-72*01
IGHV3-72*01-A
number of different entries
CDR3 length

CDR3 length of sequences associated to
the 5'UTR-leader sequence of IGHV3-73*01

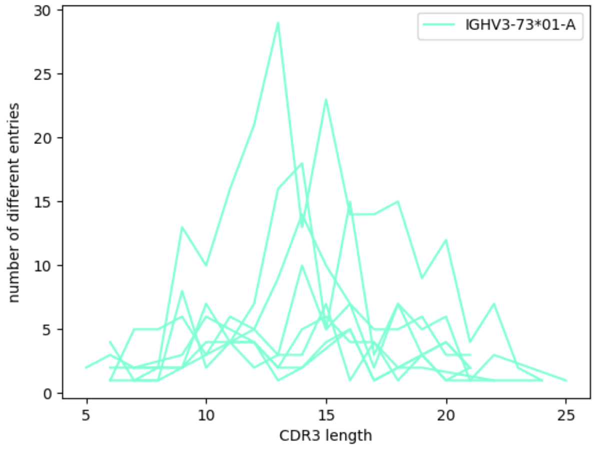

CDR3 length of sequences associated to
the 5'UTR-leader sequence of IGHV3-73*02

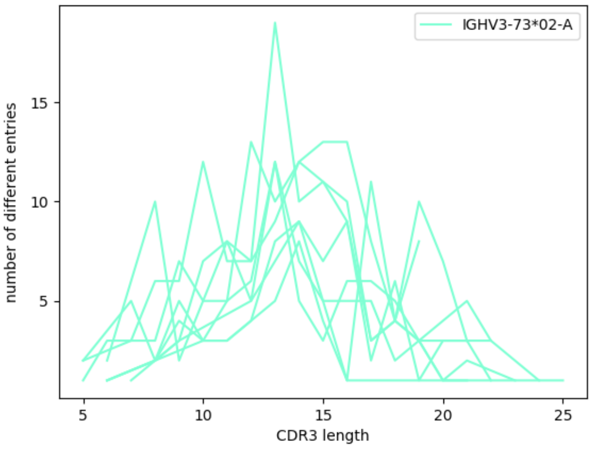

CDR3 length of sequences associated to
the 5'UTR-leader sequence of IGHV3-74*01

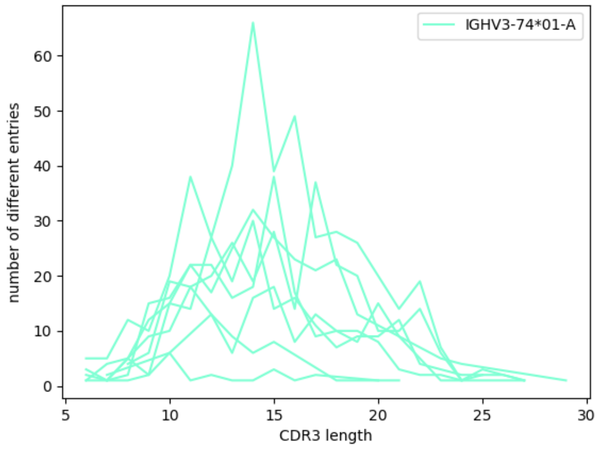

CDR3 length of sequences associated to
the 5'UTR-leader sequence of IGHV3-7*01

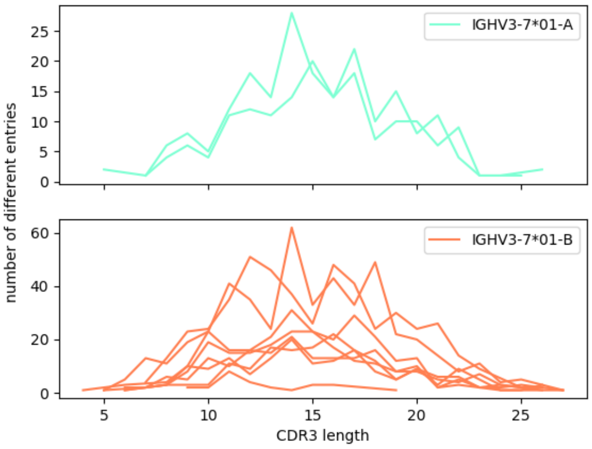

CDR3 length of sequences associated to
the 5'UTR-leader sequence of IGHV3-7*03

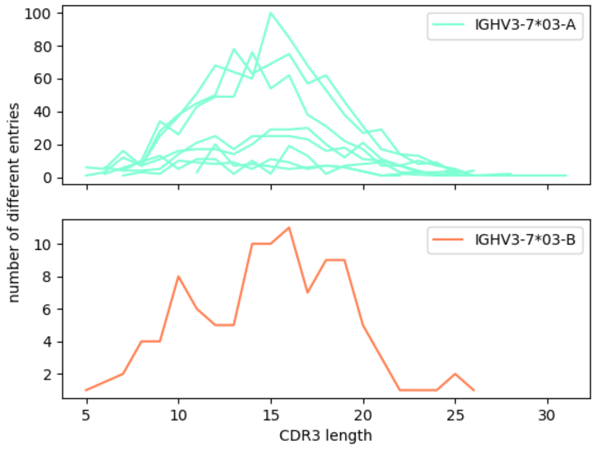

CDR3 length of sequences associated to
the 5'UTR-leader sequence of IGHV3-7*03_S9833

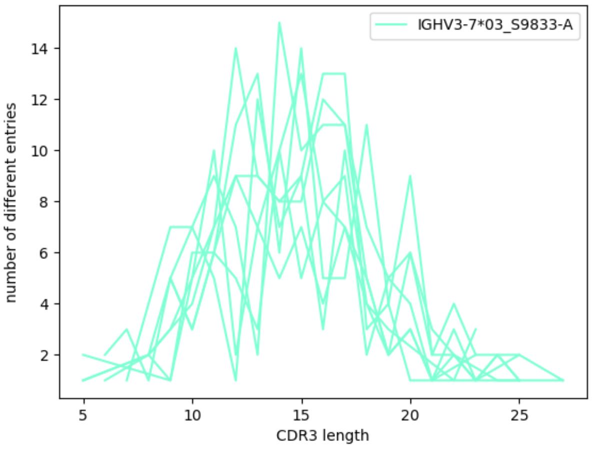

CDR3 length of sequences associated to
the 5'UTR-leader sequence of IGHV3-7*04

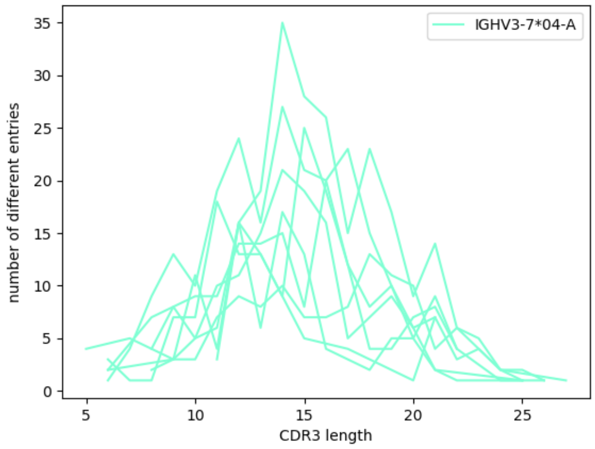

CDR3 length of sequences associated to
the 5'UTR-leader sequence of IGHV3-9*01

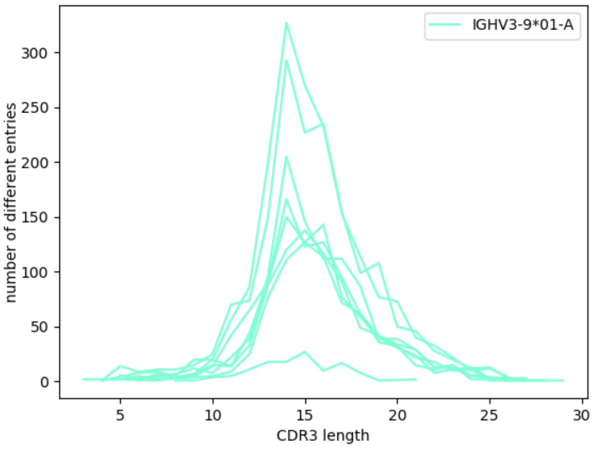

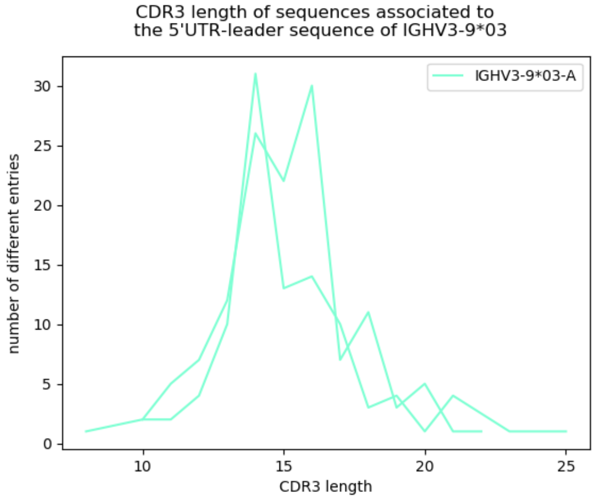
CDR3 length of sequences associated to
the 5'UTR-leader sequence of IGHV3-9*03
IGHV3-9*03-A
number of different entries
CDR3 length

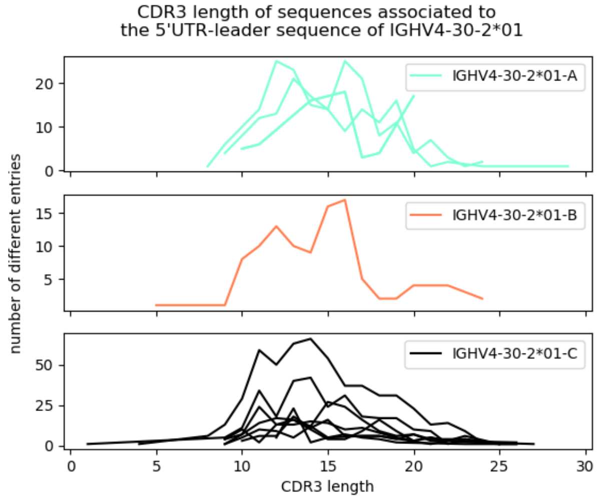
CDR3 length of sequences associated to
the 5'UTR-leader sequence of IGHV4-30-2*01
IGHV4-30-2*01-A
IGHV4-30-2*01-B
IGHV4-30-2*01-C
number of different entries
CDR3 length

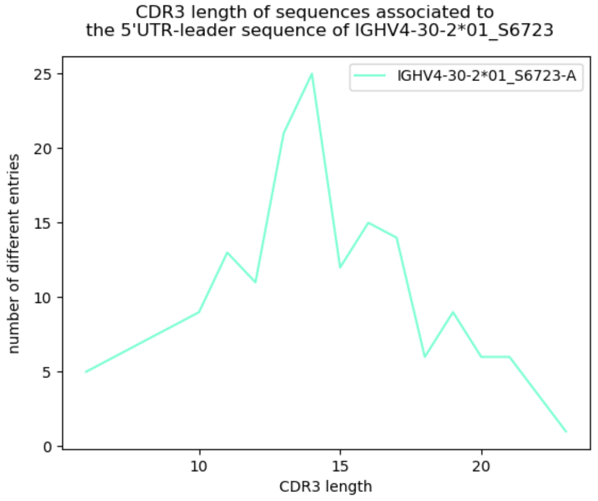
CDR3 length of sequences associated to
the 5'UTR-leader sequence of IGHV4-30-2*01_S6723
IGHV4-30-2*01_S6723-A
number of different entries
CDR3 length

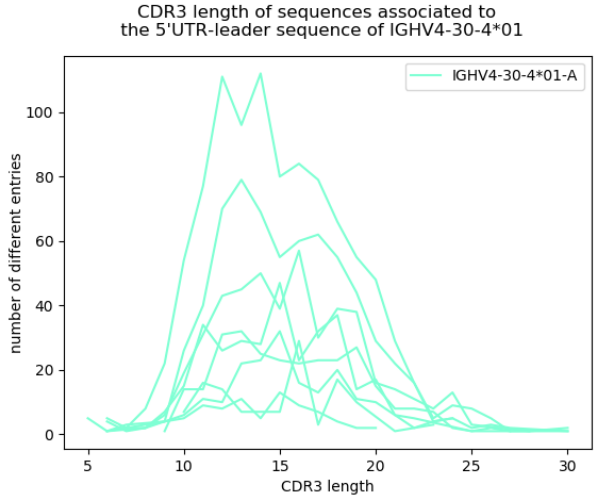
CDR3 length of sequences associated to
the 5'UTR-leader sequence of IGHV4-30-4*01
IGHV4-30-4*01-A
number of different entries
CDR3 length

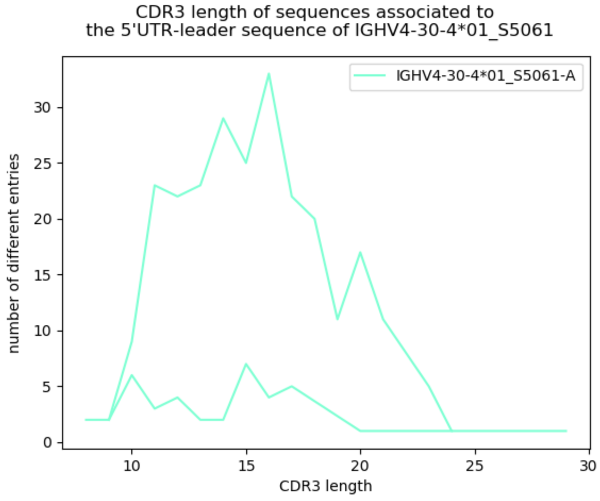
CDR3 length of sequences associated to
the 5'UTR-leader sequence of IGHV4-30-4*01_S5061
IGHV4-30-4*01_S5061-A
number of different entries
CDR3 length

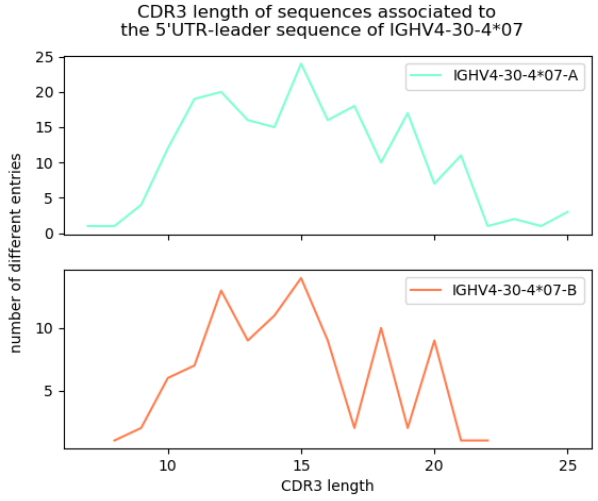
CDR3 length of sequences associated to
the 5'UTR-leader sequence of IGHV4-30-4*07
IGHV4-30-4*07-A
IGHV4-30-4*07-B
number of different entries
CDR3 length

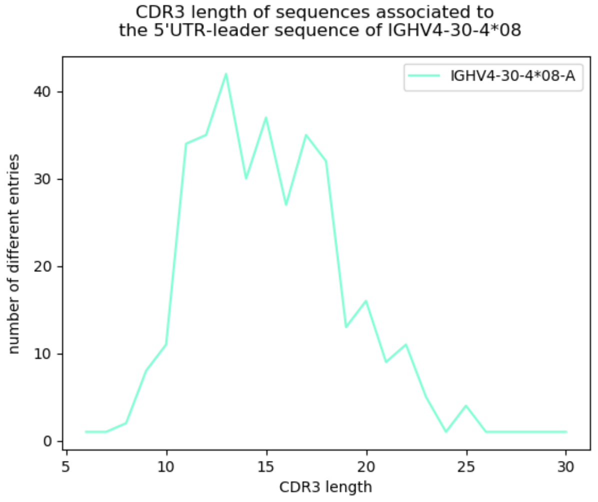
CDR3 length of sequences associated to
the 5'UTR-leader sequence of IGHV4-30-4*08
IGHV4-30-4*08-A
number of different entries
CDR3 length

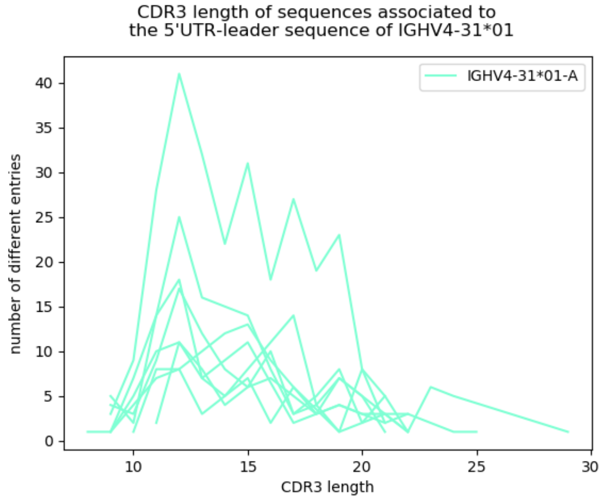
CDR3 length of sequences associated to
the 5'UTR-leader sequence of IGHV4-31*01
IGHV4-31*01-A
number of different entries
CDR3 length

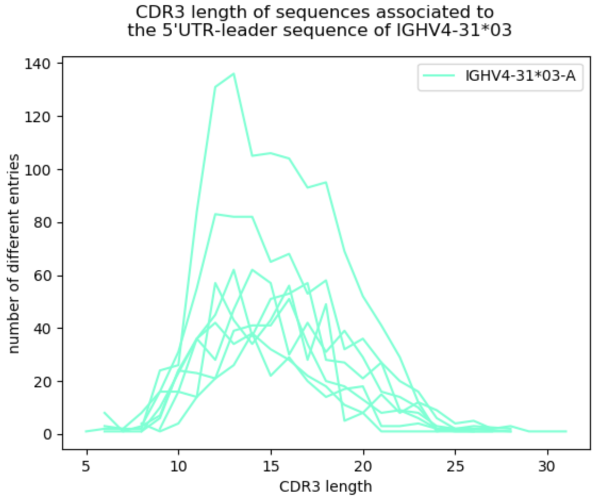
CDR3 length of sequences associated to
the 5'UTR-leader sequence of IGHV4-31*03
IGHV4-31*03-A
number of different entries
CDR3 length

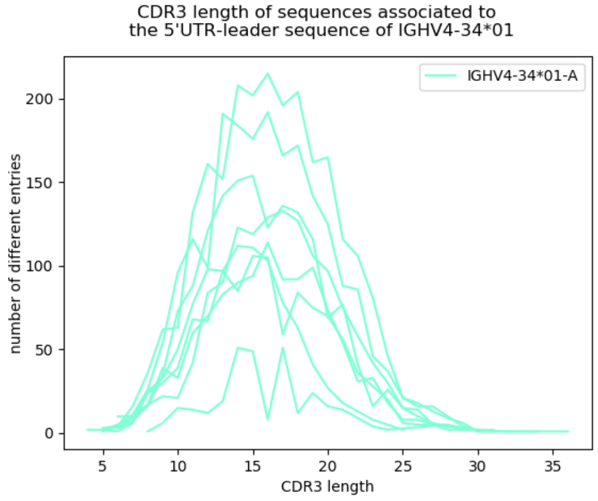
CDR3 length of sequences associated to
the 5'UTR-leader sequence of IGHV4-34*01
IGHV4-34*01-A
number of different entries
CDR3 length

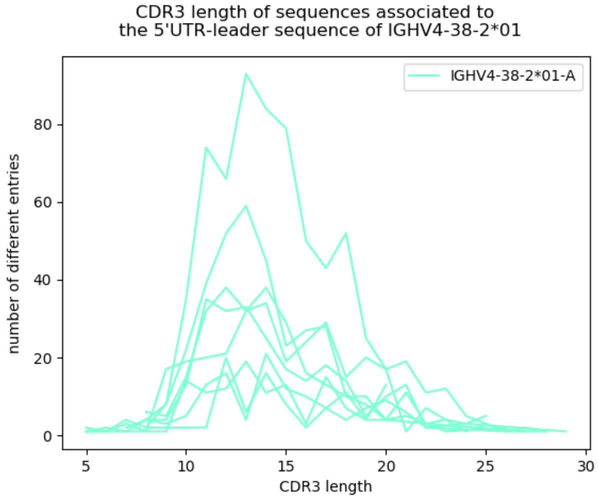
CDR3 length of sequences associated to
the 5'UTR-leader sequence of IGHV4-38-2*01
IGHV4-38-2*01-A
number of different entries
CDR3 length

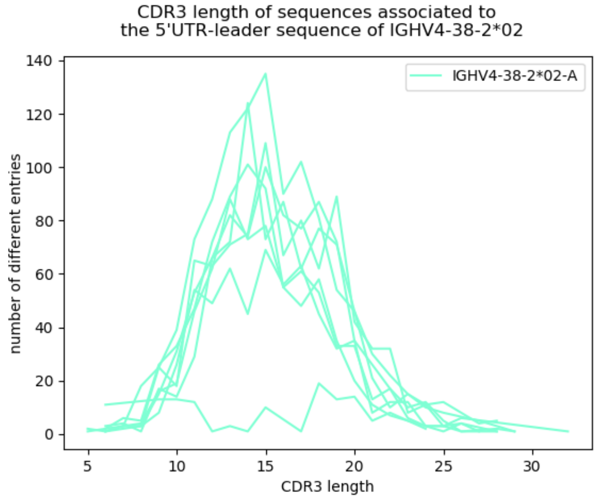
CDR3 length of sequences associated to
the 5'UTR-leader sequence of IGHV4-38-2*02
IGHV4-38-2*02-A
number of different entries
CDR3 length

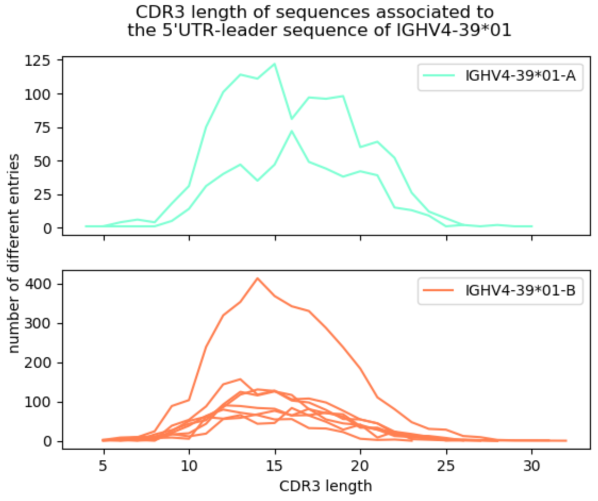
CDR3 length of sequences associated to
the 5'UTR-leader sequence of IGHV4-39*01
IGHV4-39*01-A
IGHV4-39*01-B
number of different entries
CDR3 length

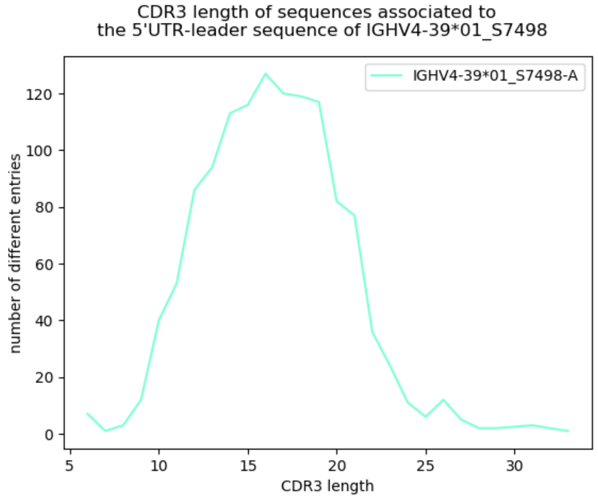
CDR3 length of sequences associated to
the 5'UTR-leader sequence of IGHV4-39*01_S7498
IGHV4-39*01_S7498-A
number of different entries
CDR3 length

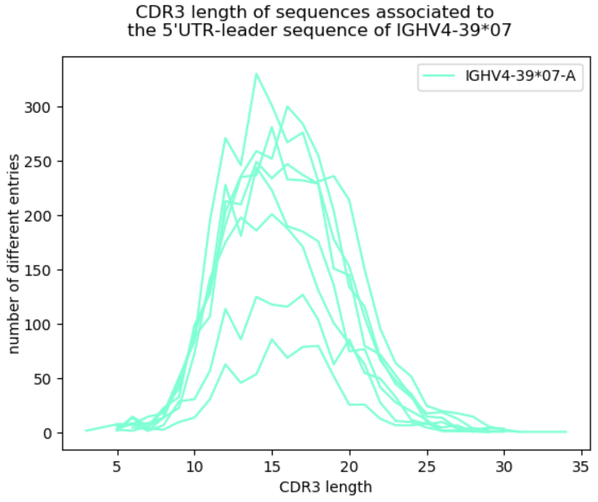
CDR3 length of sequences associated to
the 5'UTR-leader sequence of IGHV4-39*07
IGHV4-39*07-A
number of different entries
CDR3 length

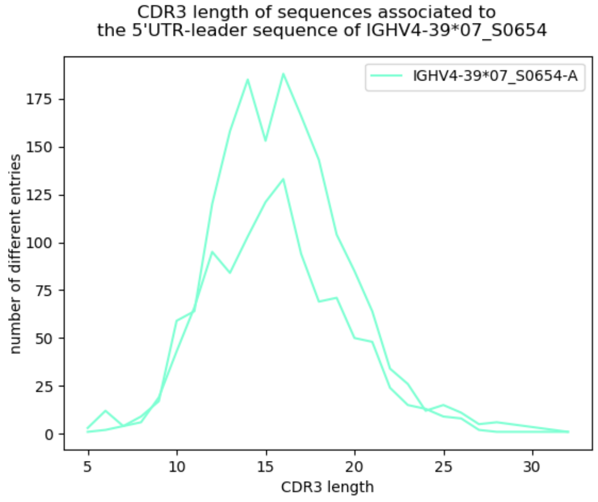
CDR3 length of sequences associated to
the 5'UTR-leader sequence of IGHV4-39*07_S0654
IGHV4-39*07_S0654-A
number of different entries
CDR3 length

CDR3 length of sequences associated to
the 5'UTR-leader sequence of IGHV4-4*02

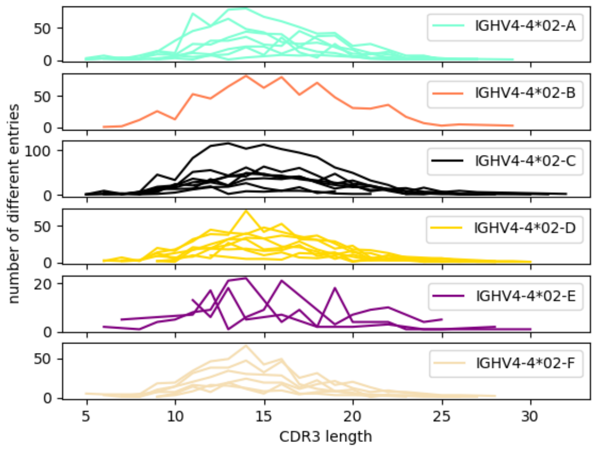

CDR3 length of sequences associated to
the 5'UTR-leader sequence of IGHV4-4*02_S2599

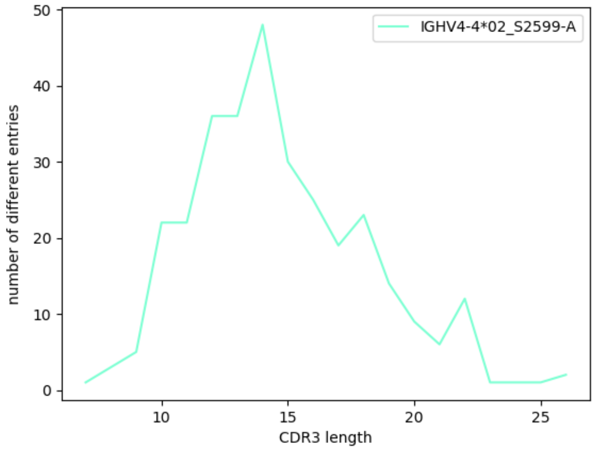

CDR3 length of sequences associated to
the 5'UTR-leader sequence of IGHV4-4*07

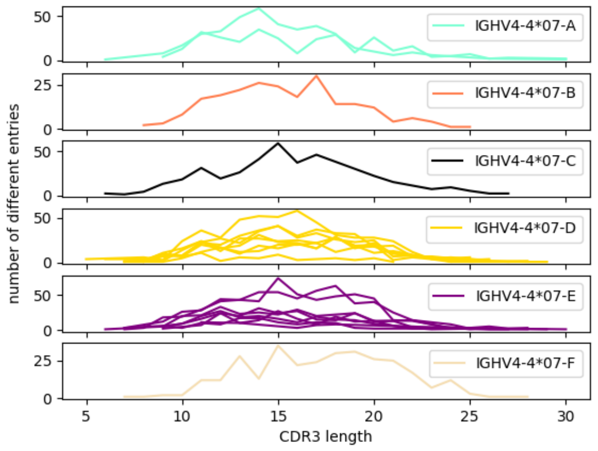

CDR3 length of sequences associated to
the 5'UTR-leader sequence of IGHV4-59*01

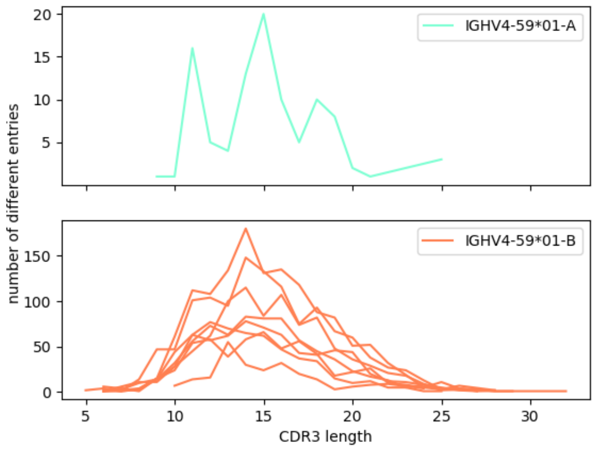

CDR3 length of sequences associated to
the 5'UTR-leader sequence of IGHV4-59*08

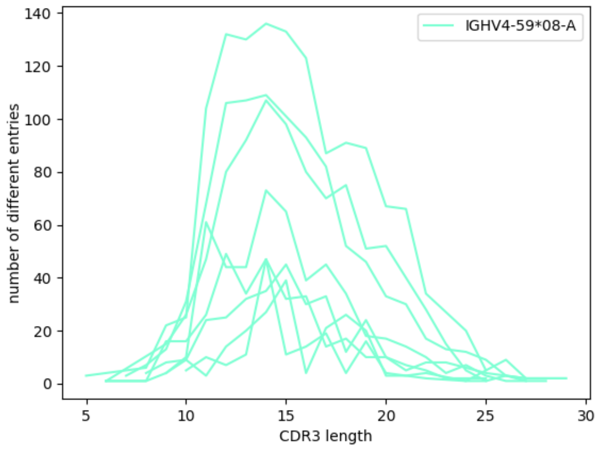

CDR3 length of sequences associated to
the 5'UTR-leader sequence of IGHV4-59*12

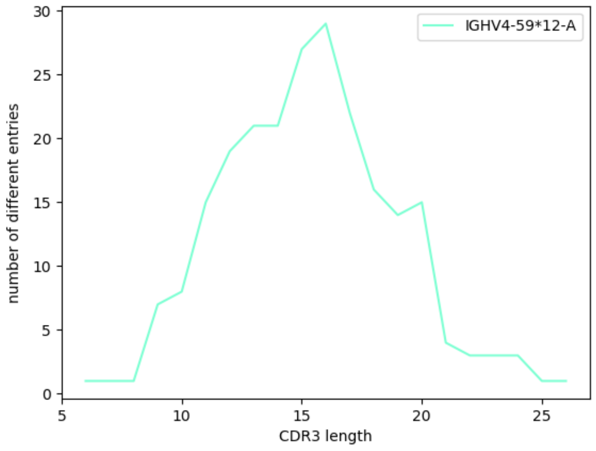

CDR3 length of sequences associated to
the 5'UTR-leader sequence of IGHV4-61*01

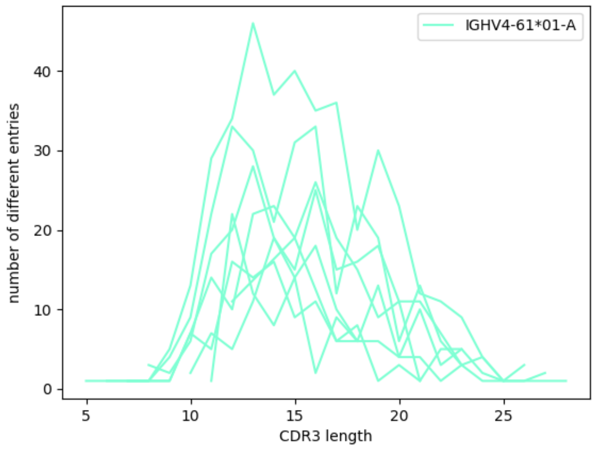

CDR3 length of sequences associated to
the 5'UTR-leader sequence of IGHV4-61*01_S9413

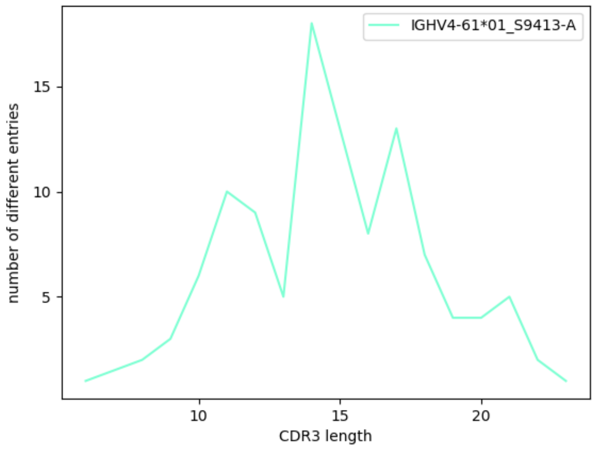

CDR3 length of sequences associated to
the 5'UTR-leader sequence of IGHV4-61*02

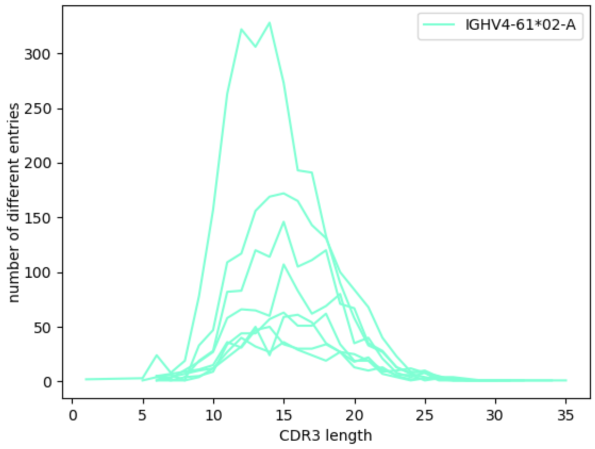

CDR3 length of sequences associated to
the 5'UTR-leader sequence of IGHV4-61*02_S0442

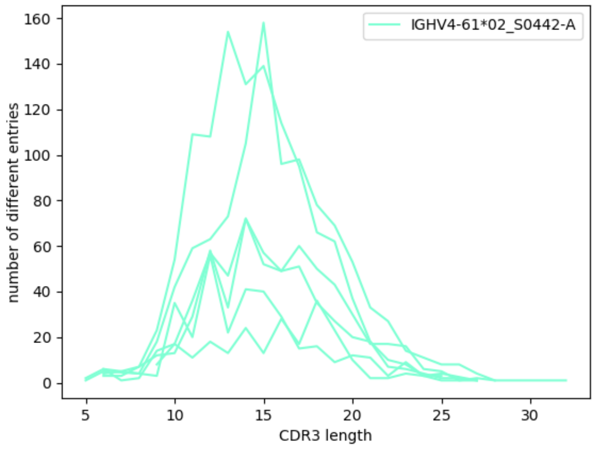

CDR3 length of sequences associated to
the 5'UTR-leader sequence of IGHV5-10-1*01

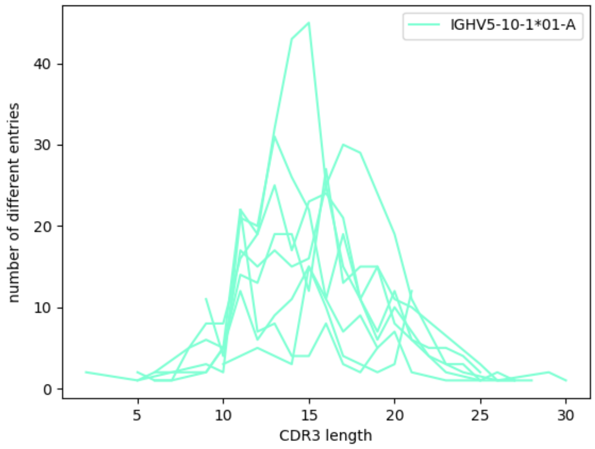

CDR3 length of sequences associated to
the 5'UTR-leader sequence of IGHV5-10-1*03

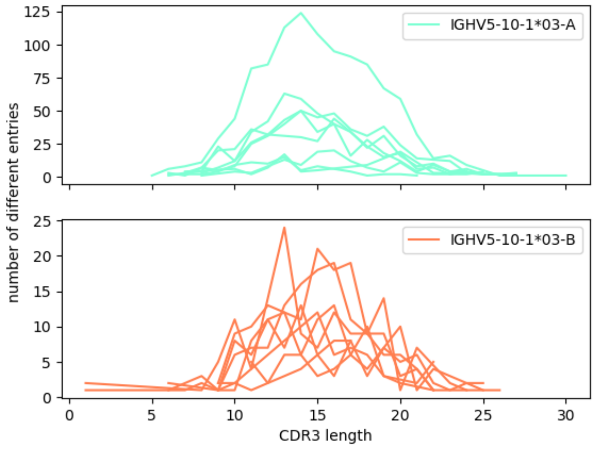

CDR3 length of sequences associated to
the 5'UTR-leader sequence of IGHV5-51*01

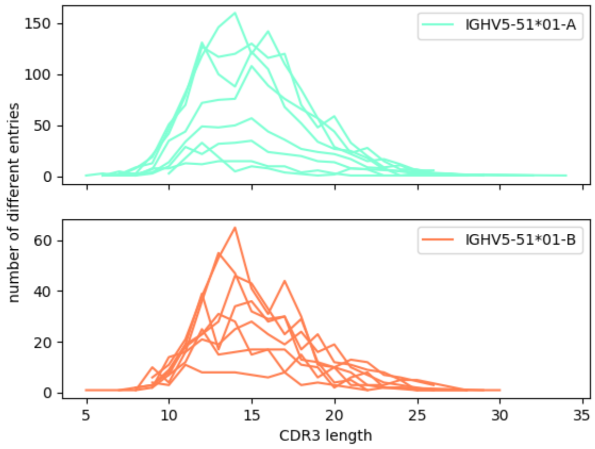

CDR3 length of sequences associated to
the 5'UTR-leader sequence of IGHV5-51*03

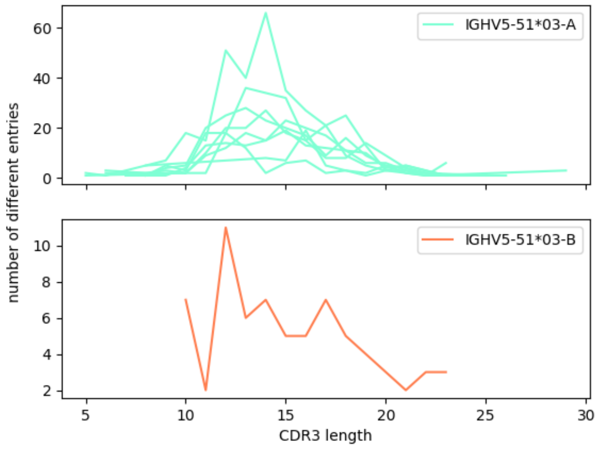

CDR3 length of sequences associated to
the 5'UTR-leader sequence of IGHV6-1*01

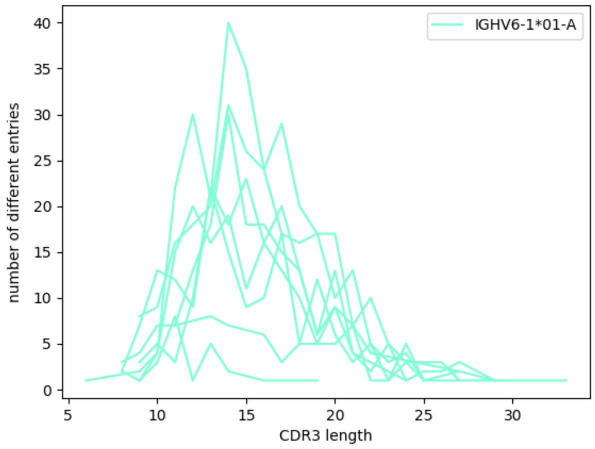

CDR3 length of sequences associated to
the 5'UTR-leader sequence of IGHV7-4-1*01

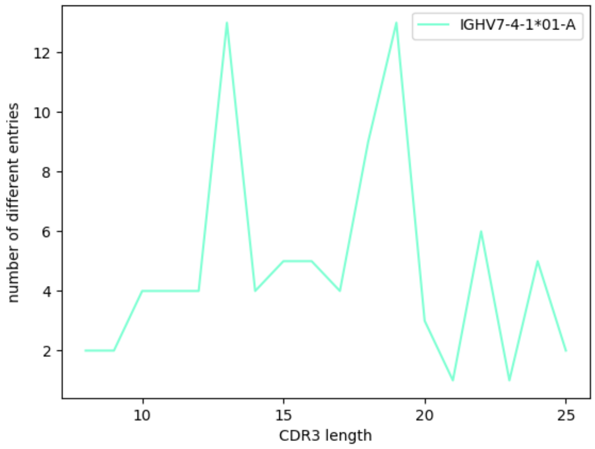

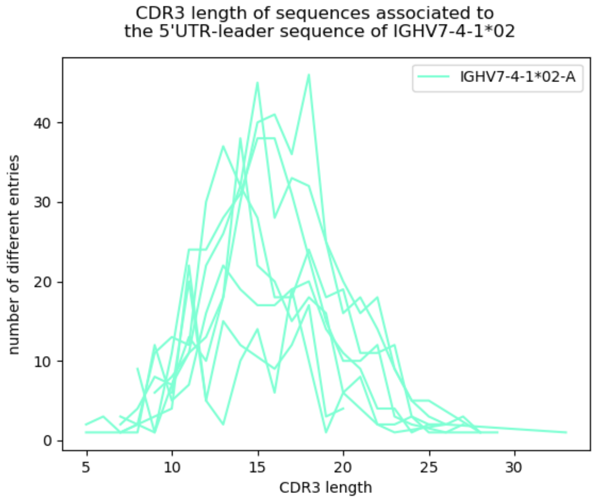

Supplementary Figure 1. Distribution patterns of CDR3 length encoded by transcripts associated to 5'UTR-leader sequences of different IGHV gene alleles. For each 5'UTR-leader sequence of a specific allele, the number of filtered reads in each length of CDR3 was counted to create the plots. Every line in the plots represents the CDR3 length distribution of one subject (at maximum 8 subjects were included in each plot).
