# Supplementary material for: Computational Inference, Validation, and Analysis of 5’UTR-Leader Sequences of Alleles of Immunoglobulin Heavy Chain Variable Genes
Source: Front Immunol. 2021 Oct 4;12:730105. doi: 10.3389/fimmu.2021.730105 (PMC8521166; doi:10.3389/fimmu.2021.730105)
Supplement: Supplementary file 1 [file DataSheet_1.zip › Supplementary information/SupplTables_1-2,4-6_SupplFig_2-10.pdf]

Supplementary information

Computational inference, validation, and analysis of 5'UTR-leader sequences of alleles of immunoglobulin heavy chain variable genes

Yixun Huang, Linnea Törnqvist and Mats Ohlin*

Dept. of Immunotechnology, Lund University, Medicon Village building 406, S-223 81 Lund, Sweden

* Corresponding author: Mats Ohlin, Dept. of Immunotechnology, Lund University, Medicon Village building 406, S-22381 LUND, Sweden. Telephone: +46-46-2224322. E-mail: mats.ohlin@immun.lth.se

Content:

Supplementary Tables 1-2, 4-6

Supplementary Figures 2-10

Supplementary Data 1 & 2, Supplementary Table 3, and Supplementary Figure 1 are provided as separate files.

Supplementary Table 1. Inference and population data of all IGHV 5'UTR-leader sequences identified in this study.

Gene	Allele and 5'UTR-leader sequence	Diversified positions	Frequency	SNPs with MAF>1% in 1000 Genomes. Overall MAF, and MAF for highest (H), lowest (L) and European (EUR) population)
IGHV1-18	01-A	-27C	2	None
	01-B	-27G	94	
	04-A	-27G	31	
IGHV1-24	01-A	-70A, -71C	2	rs8013154, -33T/C: 2% (H: 7%, L: 0%, EUR: 0%)<br>rs4511432, -71C/T: 11% (H: 31%, L: 1%, EUR: 13%)
	01-B	-70G, -71T	30	
	01-C	-70G, -71C	91	
IGHV1-2	02-A		68	None
	04-A		64	
	05-A		1	
	06-A		19	
IGHV1-3	01-A	-8T, -61C, -65G	1	rs1143496, -8C/T: 42% (H: 63%, L: 26%, EUR: 41%)<br>rs34315295, -61C/T: 27% (H: 43%, L: 15%, EUR: 15%)<br>rs56372013, -65T/G: 28% (H: 43%, L: 8%, EUR: 43%)
	01-B	-8C, -61C, -65T	9	
	01-C	-8C, -61T, -65T	26	
	01-D	-8C, -61C, -65G	57	
	01_S6816-A	-8C, -61C, -65G	2	
IGHV1-46	01-A		90	None
	03-A		49	
	04-A		2	
IGHV1-58	01-A	-39A	61	rs148981028, -24G/A: 1% (H: 5%, L: 0%, EUR: 0%)<br>rs1858692, -39A/G: 17% (H: 20%, L: 12%, EUR: 16%)
	02-A	-39A	23	
	02-B	-39G	16	
IGHV1-69-2	01-A	-70A, -71C	1	None[&]
	01-B	-70G, -71A	29	

Gene	Allele and 5'UTR-leader sequence	Diversified positions	Frequency	SNPs with MAF>1% in 1000 Genomes.<br>Overall MAF, and MAF for highest (H),<br>lowest (L) and European (EUR) population)
IGHV1-69	01-A	-88A, -100C	76	rs4448834, -88A/G: 16% (H: 28%, L: 0%, EUR: 22%)<br>rs10220412, -100G/C: 45% (H: 59%, L: 32%, EUR: 59%)<br>rs59577815, -105A/C: 2% (H: 9%, L: 0%, EUR: 0%)
	01-B	-88G, -100C	1	
	01_S8909-A	-88A, -100C	1	
	02-A	-88A, -100G	45	
	04-A	-88A, -100G	24	
	04-B	-88G, -100C	2	
	04_S3852-A	-88A, -100G	2	
	06-A	-88G, -100C	40	
	06-B	-88A, -100C	2	
	06_S0471-A	-88G, -100C	1	
	09-A	-88A, -100C	2	
	09-B	-88A, -100G	1	
	09-C	-88G, -100C	5	
	10-A	-88A, -100G	2	
	12-A	-88A, -100G	3	
	17-A	-88G, -100C	2	
IGHV1-8	01-A		77	None
	03-A		2	
IGHV2-26	01-A	-49A	98	rs2073678, -49A/G: 13% (H: 25%, L: 3%, EUR: 3%)
	02_S3803-A	-49G	1	
IGHV2-5	01-A		57	None
	02-A		82	

Gene	Allele and 5'UTR-leader sequence	Diversified positions	Frequency	SNPs with MAF>1% in 1000 Genomes. Overall MAF, and MAF for highest (H), lowest (L) and European (EUR) population)
IGHV2-70	01-A	-63T	63	rs61734101, -38C/T: 11% (H: 27%, L: 3%, EUR: 3%)<br>rs10144801, -63C/T: 47% (H: 68%, L: 23%, EUR: 68%)
	04-A	-63T	26	
	04_S5392-A	-63T	1	
	15-A	-63T	6	
	15-B	-63C	36	
IGHV3-11	01-A		76	rs74207678, -39T/C: 1% (H: 7%, L: 0%, EUR: 0%)
	04-A		2	
	05-A		22	
	06-A		47	
IGHV3-13	01-A		61	None
	01_S3164-A		8	
	04-A		19	
	05-A		22	
IGHV3-15	01-A		96	rs10141897, -125G/A: 9% (H: 31%, L: 0%, EUR: 1%)
	07-A		25	
IGHV3-20	01-A		53	rs12590474, -101C/T: 2% (H: 8%, L: 0%, EUR: 0%)
	04-A		30	
IGHV3-21	01-A	-76T	95	rs73377566, -76T/G: 8% (H: 16%, L: 0%, EUR: 8%)
	01-B	-76G	15	
	01_S4935-A	-76T	1	
	01_S5913-A	-76T	1	
IGHV3-23	01-A		97	rs112014369, -59C/T: 1% (H: 4%, L: 0%, EUR: 0%)<br>rs12436700, -61C/T: 5% (H: 10%, L: 0%, EUR: 0%)<br>rs12433853, -65A/G: 6% (H: 14%, L: 0%, EUR: 0%)
	04-A		20	
IGHV3-30-3	01-A		69	No data°

Gene	Allele and 5’UTR-leader sequence	Diversified positions	Frequency	SNPs with MAF>1% in 1000 Genomes. Overall MAF, and MAF for highest (H), lowest (L) and European (EUR) population)
IGHV3-30	01-A	-80G, -103C, -111G, -124G	11	rs371246072, -40C/G:2% (H: 9%, L: 0%, EUR: 0%) rs8021362, -103C/G: 48% (H: 62%, L: 36%, EUR: 62%)
	02-A	-80T, -103G, -111A, -124G	3	
	02-B	-80G, -103G, -111A, -124G	6	
	02_S4989-A	-80G, -103G, -111A, -124G	1	
	03-A	-80G, -103G, -111A, -124G	5	
	04-A	-80T, -103C, -111G, -124C	6	
	04-B	-80G, -103C, -111G, -124C	12	
	04_S7005-A	-80G, -103G, -111A, -124G	1	
	18-A	-80G, -103G, -111A, -124G	92	
	19_S5956-A	-80T, -103C, -111G, -124C	1	
IGHV3-33	01-A		96	rs146691759, -80G/T: 12% (H: 37%, L: 0%, EUR: 3%)
	01_S3418-A		1	
IGHV3-43D	03-A		15	No data°
	04-A		13	
	04_S5432-A		1	
IGHV3-43	01-A	-62G	73	rs61999678, -62G/T: 16% (H: 36%, L: 4%, EUR: 6%)
	02-A	-62T	11	
IGHV3-48	01-A		50	None
	02-A		65	
	03-A		34	
	04-A		10	
IGHV3-49	03-A		59	None
	04-A		37	
	05-A		60	

Gene	Allele and 5'UTR-leader sequence	Diversified positions	Frequency	SNPs with MAF>1% in 1000 Genomes. Overall MAF, and MAF for highest (H), lowest (L) and European (EUR) population)
IGHV3-53	01-A	-17T	68	rs2731152, -17T/C: 25% (H: 43%, L: 0%, EUR: 35%)
	02-A	-17C	49	
	02_S9017-A	-17C	1	
	04-A	-17T	29	
IGHV3-64D	06-A	-21A	52	No data°
	06_S5429-A	-21G	12	
	IGHV3-64*05_S2482-A§	-21A	10	
IGHV3-64	01-A		46	rs2073670, -56C/T: 42% (H: 61%, L: 24%, EUR: 61%)
IGHV3-66	01-A		42	None
	02-A		29	
	02_S8911-A		1	
IGHV3-72	01-A		47	None
IGHV3-73	01-A		51	rs61752554, -37G/C: 6% (H: 20%, L: 0%, EUR: 0%)
	02-A		64	
IGHV3-74	01-A		98	rs140191839, -70C/G: 3% (H: 10%, L: 0%, EUR: 0%)
IGHV3-7	01-A	-52A	76	rs2073679, -52A/G: 46% (H: 58%, L: 31%, EUR: 43%)
	01-B	-52G	2	
	03-A	-52G	44	
	03-B	-52A	1	
	03_S9833-A	-52G	11	
	04-A	-52G	21	
IGHV3-9	01-A		77	rs587598935, -60A/G: 8% (H: 13%, L: 2%, EUR: 13%)<br>rs377293660, -88A/G: 1% (H: 3%, L: 1%, EUR: 1%)<br>rs587754866, -101G/C: 20% (H: 30%, L: 10%, EUR: 26%)<br>rs587664283, -127G/A:1% (H: 2%, L: 0%, EUR: 1%)
	03-A		2	

Gene	Allele and 5’UTR-leader sequence	Diversified positions	Frequency	SNPs with MAF>1% in 1000 Genomes. Overall MAF, and MAF for highest (H), lowest (L) and European (EUR) population)
IGHV4-30-2	01-A	-65G, -66T, -69Del	3	No data°
	01-B	-65A, -66C, -69C	1	
	01-C	-65G, -66T, -69C	69	
	01_S6723-A	-65G, -66T, -69C	1	
IGHV4-30-4	01-A	-69C	62	No data°
	01_S5061-A	-69C	2	
	07-A	-69Del	2	
	07-B	-69C	1	
	08-A	-69C	1	
IGHV4-31	01-A		9	rs1393987777, -31G/C: 8% (H: 25%, L: 1%, EUR: 2%)<br>rs371293120, -49C/T: 8% (H: 27%, L: 1%, EUR: 2%)<br>rs112628447, -69C/Del: 1% (H: 3%, L: 0%, EUR: 0%)
	03-A		89	
IGHV4-34	01-A		98	None
IGHV4-38-2	01-A		23	No data°
	02-A		19	
IGHV4-39	01-A	-30G, -58A, -69A, -73T	2	rs4774113, -58A/C: 22% (H: 33%, L: 9%, EUR: 9%)<br>rs4774114, -69A/C: 22% (H: 33%, L: 9%, EUR: 9%)<br>rs4774115, -73T/C: 21% (H: 32%, L: 8%, EUR: 8%)
	01-B	-30C, -58A, -69A, -73T	92	
	01_S7498-A	-30C, -58A, -69A, -73T	1	
	07-A	-30C, -58C, -69C, -73C	13	
	07_S0654-A	-30C, -58C, -69C, -73C	2	

Gene	Allele and 5'UTR-leader sequence	Diversified positions	Frequency	SNPs with MAF>1% in 1000 Genomes. Overall MAF, and MAF for highest (H), lowest (L) and European (EUR) population)
IGHV4-4	02-A	-1T, -31C, -65A, -66C, -74G, -78A, -81C	7	rs78225485, -1T/C: 49% (H: 65%, L: 37%, EUR: 51%)<br>rs1141386. -31C/G: 18% (H: 32%, L: 3%, EUR: 32%)<br>rs199897176, -52A/G: 2% (H: 6%, L: 0%, EUR: 0%)<br>rs200645461, -65A/G: 5% (H: 13%, L: 2%, EUR: 2%)<br>rs201659551, -66C/T: 5% (H: 13%, L: 2%, EUR: 2%)<br>rs77360520, -74A/G: 18% (H: 40%, L: 5%, EUR: 5%)<br>rs56086041, -78A/C: 31% (H: 61%, L: 13%, EUR: 17%)<br>rs55998743, -81C/G: 34% (H: 65%, L: 14%, EUR: 18%)
	02-B	-1T, -31C, -65A, -66C, -74A, -78A, -81G	1	
	02-C	-1T, -31C, -65A, -66C, -74A, -78A, -81C	57	
	02-D	-1T, -31C, -65A, -66C, -74A, -78C, -81G	22	
	02-E	-1T, -31C, -65G, -66T, -74A, -78A, -81C	3	
	02-F	-1C, -31C, -65A, -66C, -74A, -78A, -81C	6	
	02_S2599-A	-1T, -31C, -65A, -66C, -74A, -78C, -81G	1	
	07-A	-1C, -31C, -65A, -66C, -74A, -78C, -81G	2	
	07-B	-1C, -31G, -65A, -66C, -74A, -78C, -81G	1	
	07-C	-1C, -31G, -65G, -66T, -74A, -78C, -81G	1	
	07-D	-1C, -31C, -65A, -66C, -74A, -78A, -81C	8	
	07-E	-1C, -31G, -65A, -66C, -74A, -78A, -81C	55	
	07-F	-1C, -31C, -65G, -66T, -74A, -78A, -81C	1	
IGHV4-59	01-A	-22G, -34T, -49T, -65G, -66T, -78C, -81G	1	rs56139643, -34T/C: 9% (H: 19%, L: 5%, EUR: 7%)
	01-B	-22A, -34T, -49T, -65G, -66T, -78C, -81G	98	
	08-A	-22A, -34T, -49T, -65G, -66T, -78C, -81G	42	
	12-A	-22A, -34C, -49C, -65A, -66C, -78A, -81C	1	
IGHV4-61	01-A	-49C	63	rs201399631, -1C/T: 11% (H: 21%, L: 3%, EUR: 3%)<br>rs2516897, -49C/T: 26% (H: 54%, L: 15%, EUR: 15%)<br>rs200506032, -65G/A: 11% (H: 22%, L: 3%, EUR: 3%)<br>rs201574166, -66T/C: 11% (H: 22%, L: 3%, EUR: 3%)
	01_S9413-A	-49C	1	
	02-A	-49T	30	
	02_S0442-A	-49T	6	
IGHV5-10-1	01-A	-35G, -37T	22	None[&]
	03-A	-35C, -37C	43	
	03-B	-35G, -37T	14	

Gene	Allele and 5'UTR-leader sequence	Diversified positions	Frequency	SNPs with MAF>1% in 1000 Genomes. Overall MAF, and MAF for highest (H), lowest (L) and European (EUR) population)
IGHV5-51	01-A	-37C, -102T	87	rs116300992, -18C/A: 1% (H: 4%, L: 0%, EUR: 0%)<br>rs61732461, -37C/T: 2% (H: 5%, L: 0%, EUR: 5%)<br>rs77785266, -102T/C: 6% (H: 15%, L: 2%, EUR: 2%)
	01-B	-37T, -102T	12	
	03-A	-37C, -102T	40	
	03-B	-37C, -102C	1	
IGHV6-1	01-A		95	None
IGHV7-4-1	01-A		1	None[&]
	02-A		26	

[&] The gene is not featured in GRCh37, and was thus analyzed using Ensembl (28) with GRCh38 as the reference genome.

° The gene is not featured in GRCh37 or GRCh38, and could thus not be analyzed.

§ Have now been included in IMGT database as IGHV3-64D*09 (Supplementary Table 3).

Supplementary Table 2. Haplotyping of distribution of reads associated to particular upstream regions of genes of the IGHV locus.

Subject	IGHV gene and upstream region	IGHJ6 read distribution	
		IGHJ6*02	IGHJ6*03
ERR2567187	IGHV1-3*01-D	72	0
	IGHV1-3*01-C	0	76
ERR2567204	IGHV1-3*01-B	160	0
	IGHV1-3*01-C	0	55
ERR2567243	IGHV1-3*01-C	48	0
	IGHV1-3*01-D	1	47
ERR2567249	IGHV1-3*01-C	1	44
	IGHV1-3*01-D	54	0
ERR2567266	IGHV1-3*01-D	78	0
	IGHV1-3*01-B	0	50
ERR2567271	IGHV1-3*01-C	28	0
	IGHV1-3*01-D	0	23
ERR2567189	IGHV1-24*01-C	31	0
	IGHV1-24*01-B	0	9
ERR2567206	IGHV1-24*01-C	0	12
	IGHV1-24*01-A	15	0
ERR2567226	IGHV1-24*01-C	0	8
	IGHV1-24*01-B	9	0
ERR2567230	IGHV1-24*01-C	50	0
	IGHV1-24*01-B	0	8
ERR2567246	IGHV1-24*01-C	49	0
	IGHV1-24*01-B	0	5
ERR2567263	IGHV1-24*01-C	54	0
	IGHV1-24*01-B	1	29
ERR2567271	IGHV1-24*01-C	12	0
	IGHV1-24*01-B	0	4
ERR2567277	IGHV1-24*01-B	0	19
	IGHV1-24*01-C	18	0
ERR2567206	IGHV1-58*02-A	20	0
	IGHV1-58*02-B	0	3
ERR2567243	IGHV1-58*02-A	11	0
	IGHV1-58*02-B	0	2
ERR2567187	IGHV1-58*02-A	0	14
	IGHV1-58*02-B	9	0
ERR2567264	IGHV1-69*06-B	2	231
	IGHV1-69*06-A	1	204
	IGHV1-69*02-A	95	0
ERR2567220	IGHV2-70*15-A	24	1
	IGHV2-70*15-B	0	18
ERR2567192	IGHV3-21*01-A	0	15
	IGHV3-21*01-B	19	0
ERR2567266	IGHV3-21*01-A	0	53
	IGHV3-21*01-B	64	1

Subject	IGHV gene and upstream region	IGHJ6 read distribution	
		IGHJ6*02	IGHJ6*03
ERR2567266	IGHV4-4*02-C	58	0
	IGHV4-4*07-A	1	107
ERR2567189	IGHV4-4*02-F	0	24
	IGHV4-4*07-E	37	0
ERR2567200	IGHV4-4*02-C	0	46
	IGHV4-4*07-B	48	0
ERR2567230	IGHV4-4*02-A	0	38
	IGHV4-4*07-D	72	0
ERR2567192	IGHV4-4*02-C	16	0
	IGHV4-4*02-A	0	17
ERR2567204	IGHV4-4*02-C	74	0
	IGHV4-4*02-D	0	75
ERR2567246	IGHV4-4*02-F	0	36
	IGHV4-4*02-C	65	0
ERR2567254	IGHV4-4*02-C	55	0
	IGHV4-4*02-F	0	42
ERR2567261	IGHV4-4*02-C	99	0
	IGHV4-4*02-F	0	78
ERR2567271	IGHV4-4*02-D	51	0
	IGHV4-4*02-F	0	5
ERR2567274	IGHV4-4*02-E	24	0
	IGHV4-4*02-C	0	21
ERR2567187	IGHV4-4*02-C	65	0
	IGHV4-4*01§	-	-
ERR2567201	IGHV4-4*07-E	27	0
	IGHV4-4*07-D	0	35
ERR2567263	IGHV4-4*07-F	0	94
	IGHV4-4*07-C	84	1
ERR2567215	IGHV4-30-2*01-C	0	39
	IGHV4-30-2*01-A	31	0
ERR2567254	IGHV4-39*01-A	0	190
	IGHV4-39*01-B	118	0
ERR2567220	IGHV5-51*01-B	25	0
	IGHV5-51*01-A	0	36
ERR2567231	IGHV5-51*01-B	0	35
	IGHV5-51*01-A	48	0
ERR2567264	IGHV5-51*01-A	2	52
	IGHV5-51*01-B	41	0
ERR2567266	IGHV5-51*01-A	72	0
	IGHV5-51*01-B	0	35

§ IGHV4-4*01 was not identified in the present IgDiscover analysis, but in a recent study emoplying IMGT/HighV-quest analysis (23).

Supplementary Table 4. 5’-end of the 5’UTR-leader sequence of sets of genes inferred in the present study, 5’-end of the 5’UTR-leader sequence as previously defined as having variants with a 5’-terminal G, and population data (Ensembl release 103) (28) corresponding to the 5’-end of the 5’-UTR, and the extent of support for determination of diverse 5’-ends of 5’UTR-leader sequence. Only data related to genes represented in the Ensembl database are shown.

Gene/allele[§]	5’end of inferred 5’UTR (this study)[†]	5’end of inferred 5’UTR by Mikocziova et al. (18)[†]	Dominant base of SNP corresponding to the 5’-base inferred by Mikocziova et al. (18); (Ensembl; irrespective of allele)	Support in population data for different 5’-bases inferred by Mikocziova et al. (18)	
IGHV1-3*01	5’-A$_{-101}$ACC	5’-C$_{-102}$AACC and 5’-G$_{-102}$AACC	C$_{-102}$ (Highest population MAF<0.01%)	5’-C$_{-102}$: yes	5’-G$_{-102}$: no
IGHV1-46*01	5’-A$_{-115}$TCA	5’-C$_{-116}$ATCA and 5’-G$_{-116}$ATCA	C$_{-116}$ (Highest population MAF<0.01%)	5’-C$_{-116}$: yes	5’-G$_{-116}$: no
IGHV1-46*03	5’-A$_{-115}$TCA	5’-C$_{-116}$ATCA and 5’-G$_{-116}$ATCA			
IGHV2-5*01	5’-A$_{-73}$CT	5’-T$_{-75}$GACT and 5’-G$_{-75}$GACT	T$_{-75}$ (Highest population MAF<0.01%)	5’-T$_{-75}$: yes	5’-G$_{-75}$: no
IGHV2-5*02	5’-A$_{-73}$CT	5’-T$_{-75}$GACT and 5’-G$_{-75}$GACT			
IGHV2-70*01	5’-A$_{-73}$AT	5’-T$_{-75}$GAAT and 5’-G$_{-75}$GAAT	T$_{-75}$ (Highest population MAF<0.01%)	5’-T$_{-75}$: yes	5’-G$_{-75}$: no
IGHV2-70*04	5’-A$_{-73}$AT	5’-T$_{-75}$GAAT and 5’-G$_{-75}$GAAT			
IGHV2-70*15	5’-A$_{-73}$AT	5’-G$_{-75}$GAAT			
IGHV3-11*01	5’-A$_{-137}$GTC	5’-C$_{-138}$AGCT and 5’-G$_{-138}$AGCT	C$_{-138}$ (Highest population MAF<0.01%)	5’-C$_{-138}$: yes	5’-G$_{-138}$: no
IGHV3-11*05	5’-A$_{-137}$GTC	5’-G$_{-138}$AGCT			
IGHV3-11*06	5’-A$_{-137}$GTC	5’-C$_{-138}$AGCT and 5’-G$_{-138}$AGCT			
IGHV3-20*01	5’-A$_{-137}$GCT	5’-G$_{-138}$AGCT	C$_{-138}$ (rs560966965; Highest population MAF (T): 4%)	5’-C$_{-138}$: yes	5’-G$_{-138}$: no
IGHV3-20*01 C307T (*04)	5’-A$_{-137}$GCT	5’-G$_{-138}$AGCT			
IGHV3-43*01	5’-A$_{-137}$GCTCT*	5’-G$_{-136}$CTCT	G (Highest population MAF<0.01%)		5’-G$_{-136}$: yes
IGHV3-43*02	5’-A$_{-137}$GCTCT*	5’-G$_{136}$CTCT			
IGHV3-53*01	A$_{-127}$GA	5’-G$_{-131}$GGGAGA	T (Highest population MAF<0.01%)	5’-T$_{-131}$: yes	5’-G$_{-131}$: no
IGHV3-53*02	G$_{-126}$A	5’-G$_{-131}$GGGAGA and 5’-T$_{-131}$GGGAGA			
IGHV3-53*04	A$_{-127}$GA	5’-G$_{-131}$GGGAGA			
IGHV3-64*01	G$_{-136}$CT	5’-C$_{-138}$AGCT and 5’-G$_{-138}$AGCT	C (Highest population MAF<0.01%)	5’-C$_{-138}$: yes	5’-G$_{-138}$: no
IGHV3-66*01	5’-A$_{-136}$GCT	5’-C$_{-137}$AGCT and 5 ’-G$_{-137}$AGCT	C (Highest population MAF<0.01%)	5’-C$_{-137}$: yes	5’-G$_{-137}$: no
IGHV3-66*02	5’-A$_{-136}$GCT	5’-G$_{-137}$AGCT			
IGHV3-72*01	5’-A$_{-130}$GAGC	5’-G$_{-138}$AGCTCTGAGAGC	C$_{-138}$ (Highest population MAF<0.01%)	5’-C$_{-138}$: yes	5’-G$_{-138}$: no

Gene/allele[§]	5’end of inferred 5’UTR (this study)[†]	5’end of inferred 5’UTR by Mikocziova et al. (18)[†]	Dominant base of SNP corresponding to the 5’-base inferred by Mikocziova et al. (18); (Ensembl; irrespective of allele)	Support in population data for different 5’-bases inferred by Mikocziova et al. (18)	
IGHV3-73*01	5’-A$_{-137}$GCTC	5’-C$_{-138}$AGCT and 5’-G$_{-138}$AGCT	C$_{-138}$ (Highest population MAF<0.01%)	5’-C$_{-138}$: yes	5’-G$_{-138}$: no
IGHV3-73*02	5’-A$_{-137}$GCTC	5’-C$_{-138}$AGCT and 5’-G$_{-138}$AGCT			
IGHV4-4*02	5’-A$_{-94}$TACTT or 5’-T$_{-93}$ACTT or 5’-A$_{-92}$CTT**	5’-T$_{-93}$ACTT and 5’-G$_{-93}$ACTT	T (rs531656003; highest population MAF (A): 1%)	5’-T$_{-93}$: yes	5’-G$_{-93}$: no
IGHV4-4*07	5’-A$_{-94}$TACTT or 5’-A$_{-92}$CTT**	5’-T$_{-93}$ACTT and 5’-G$_{-93}$ACTT			
IGHV4-31*03	5’-A$_{-94}$TACTT	5’-T$_{-93}$ACTT and 5’-G$_{-93}$ACTT	T$_{-93}$ (Highest population MAF<0.01%)	5’-T$_{-93}$: yes	5’-G$_{-93}$: no
IGHV4-34*01	5’-A$_{-95}$GTGCTTT***	5’-G$_{-92}$CTTT	G$_{-92}$		5’-G$_{-92}$: yes
IGHV4-59*01	5’-A$_{-92}$CTT	5’-T$_{-93}$ACTT and 5’-G$_{-93}$ACTT	T$_{-93}$ (Highest population MAF<0.01%)	5’-T$_{-93}$: yes	5’-G$_{-93}$: no
IGHV4-59*08	5’-A$_{-92}$CTT	5’-T$_{-93}$ACTT and 5’-G$_{-93}$ACTT			
IGHV4-61*01	5’-A$_{-92}$CTT	5’-T$_{-93}$ACTT and 5’-G$_{-93}$ACTT	T$_{-93}$ (Highest population MAF<0.01%)	5’-T$_{-93}$: yes	5’-G$_{-93}$: no
IGHV4-61*02	5’-A$_{-92}$CTT	5’-T$_{-93}$ACTT and 5’-G$_{-93}$ACTT			
IGHV6-1*01	5’-A$_{-110}$GAG	5’-C$_{-111}$AGAG and 5’-G$_{-111}$AGAG	C$_{-111}$ (Highest population MAF<0.01%)	5’-C$_{-111}$: yes	5’-G$_{-111}$: no

§ Genes/alleles that feature a 5’G in (some of) its inferred 5’UTR-leader sequences and that are featured with population data in the Ensemble database.
† The 5’-most base is numbered as the number of bases upstream of the first base encoding the H chain variable domain
* Additional 5’-A inferred in this study is supported by genomic data
** Different lengths determined depending on which 5’UTR-leader was inferred
*** Additional 5’-AGT inferred in this study is supported by genomic data

Supplementary Table 5. Lack of evidence for inference of base -93 in upstream regions of IGHV4 alleles. This base is close to the limit of called bases in reads representing upstream regions of genes belonging to this subgroup. The ratio should be close to 100% or 0% if evidence of G or T, respectively, at position -93 is present among the transcripts. Haplotype distribution of base -93, as defined by association to different alleles of IGHJ6, was calculated from the reads precisely matching the inferred 5'UTR-leader sequence from base -92 to -1, and unequivocally assigned by the IMGT/HighV-Quest tool (17) to the allele in question. The call of base -93 of alleles of IGHV1-46, a gene whose readable upstream region commonly extends beyond base -93, is shown for comparison, a call that with high confidence identify T as the base found in position -93.

Dataset	Gene	Allele	5'UTR-leader	IGHV6*02 base -93: G / (T+G) (%)	IGHV6*03 base -93: G / (T+G) (%)
ERR2567187	IGHV4-30-2	*01	C	54	51
	IGHV4-30-4	*01	A	24	27
	IGHV4-31	*03	A	39	32
	IGHV4-34	*01	A	33	34
	IGHV4-38-2	*01	A	67	
	IGHV4-39	*01	B		58
	IGHV4-4	*01			†
		*02	C	43	
	IGHV4-59	*01	B	46	53
		*08	A		45
	IGHV4-61	*02	A	78	
	IGHV1-46	*01	A	1	1
ERR2567189	IGHV4-30-2	*01	C	52	
	IGHV4-30-4	*01	A	43	
	IGHV4-31	*03	A	33	
	IGHV4-34	*01	A	28	35
	IGHV4-38-2	*02	A	60	
	IGHV4-39	*01	B	60	54
	IGHV4-4	*02	F		37
		*07	E	48	
	IGHV4-59	*01	B	47	43
		*08	A	50	
	IGHV4-61	*01[∞]	A		43
	IGHV1-46	*01	A		3
		*03	A	0	
ERR2567192	IGHV4-30-2	*01	C		69
	IGHV4-30-4	*01[∞]	A		47
	IGHV4-31	*03	A	41	0
	IGHV4-34	*01	A	37	26
	IGHV4-39	*01	B	50	73
	IGHV4-4	*02	C	18	
			A		14
	IGHV4-59	*01	B	25	46
		*08	A	13	30
	IGHV1-46	*01	A	8	0
ERR2567199	IGHV4-31	*03	A		41
	IGHV4-34	*01	A	36	32
	IGHV4-38-2	*02	A	70	
	IGHV4-39	*01	B		59
		*07	A	63	
	IGHV4-4	*02	C		54
		*07	E	36	
	IGHV4-59	*01	B	43	50
	IGHV4-61	*01[∞]	A	58	40
	IGHV1-46	*01	A	2	0

Dataset	Gene	Allele	5'UTR-leader	IGHV6*02<br>base -93: G / (T+G) (%)	IGHV6*03<br>base -93: G / (T+G) (%)
ERR2567200	IGHV4-30-2	*01	C	49	
	IGHV4-31	*01[∞]	A		34
		*03	A	36	
	IGHV4-34	*01	A	30	34
	IGHV4-38-2	*01	A		68
	IGHV4-39	*01	B	57	
	IGHV4-4	*02	C		34
		*07	B	59	
	IGHV4-59	*01	B	46	52
	IGHV4-61	*01[∞]	A		35
		*01__S9413 (A41G)	A	43	
	IGHV1-46	*01	A		0
		*03	A	2	
ERR2567201	IGHV4-30-2	*01	C	67	41
	IGHV4-30-4	*01	A	38	27
	IGHV4-31	*03	A	57	37
	IGHV4-34	*01	A	37	33
	IGHV4-39	*01	B	56	52
	IGHV4-4	*07	E	27	
			D		36
	IGHV4-59	*01	B	52	49
	IGHV4-61	*01[∞]	A		47
		*02	A	31	
	IGHV1-46	*01	A		3
		*03	A	1	
ERR2567204	IGHV4-30-2	*01	C	55	
	IGHV4-31	*03	A		37
	IGHV4-34	*01	A	35	38
	IGHV4-39	*01	B	64	58
	IGHV4-4	*02	C	46	
			D		53
	IGHV4-59	*01	B	54	46
	IGHV4-61	*01[∞]	A	37	
		*02	A		68
	IGHV1-46	*01	A	1	
		*03	A		4
ERR2567206	IGHV4-30-2	*01	C		50
	IGHV4-34	*01	A	31	27
	IGHV4-39	*01	B		52
		*07_S0654 (C288A)	A	62	
	IGHV4-4	*01		†	
		*02	F		55
	IGHV4-59	*01	B	50	45
		*08	A	44	
	IGHV4-61	*02	A		67
	IGHV1-46	*01	A	6	
		*03	A		4

[†] IGHV4-4*01, a very poorly expressed allele not directly studied in the present investigation, is present in this haplotype (23).

[∞] IGHV4-31*01 and IGHV4-61*01 are poorly expressed in comparison to IGHV4-31*03 and IGHV4-61*02, respectively, and may not be inferred depending on inference software settings.

Supplementary Table 6. Inference and population data suggesting that upstream regions of primary IMGT entries, in addition to IGHV2-5*01 (Supplementary Figure 5), may be in error.

Germline gene	Inferred upstream sequences and sequences defined by primary IMGT entries*	Population data (Ensembl Genome Browser)
IGHV1-18*01	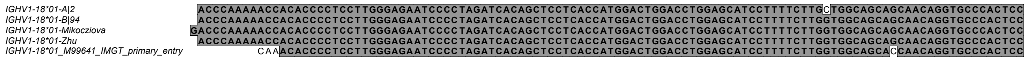	Expected base at -18 is G. Highest population MAF for this base: <0.01%
IGHV1-8*01	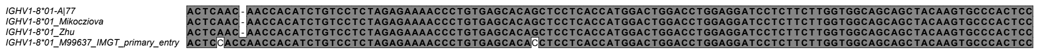	Expected base at -67 is G. Highest population MAF for this base: <0.01%
IGHV3-11*01	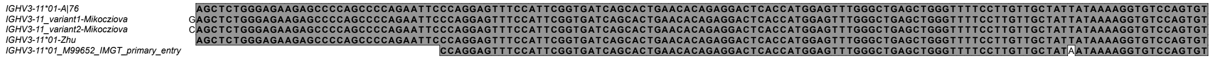	Expected base at -19 is T. Highest population MAF for this base: <0.01%
IGHV3-23*01	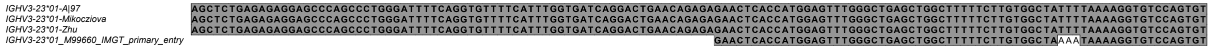	Expected bases at -20 – -18 are TTT. Highest population MAF for these bases: <0.01%
IGHV3-64*01	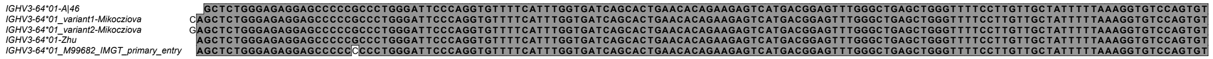	Expected base at -116 is G. Highest population MAF for this base: <0.01%
IGHV3-72*01	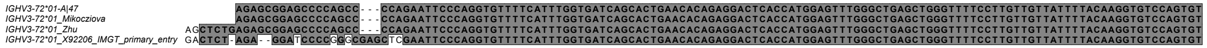	Expected base for instance at bases -111 – -112 are AC. Highest population MAF for these bases: <0.01%
IGHV4-4*02	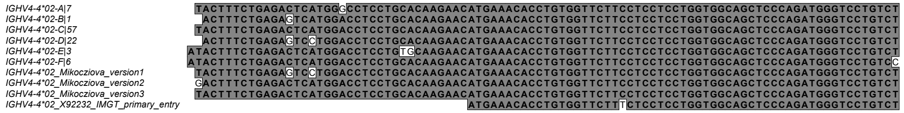	Expected base at -37 is C. Highest population MAF for this base: <0.01%
IGHV5-51*01	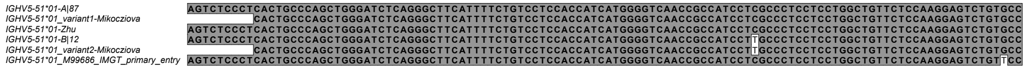	Expected base at -3 is G. Highest population MAF for this base: <0.01%

* Inferred upstream regions were defined in this study, by Mikocziova et al. (18) and Zhu et al. (14). In the latter case only the most frequent variants were included and major variants of IGHV4-4*02 upstream regions defined by Zhu et al are shown in Supplementary Figure 3..

```
IGHV3-43D*03/*04/*04_S5432  AGCTCTGGGAAAGGAGCCCCAGCCCTGAGATTCCCAGGTGTTTCCATTCGGTGATCAGCACTGAACACAGA--ACTCACCATGGAGTTTGGACTGAGCTGGGTTTTCCTTGTTGCTATTTTAAAAGGTGTCCAGTGT
IGHV3-43*01                 AGCTCTGGGAGAGGAGCCCCAGCCCTGAGATTCCCAGGTGTTTCCATTCGGTGATCAGCACTGAACACAGAGAACGCACCATGGAGTTTGGACTGAGCTGGGTTTTCCTTGTTGCTATTTTAAAAGGTGTCCAGTGT
IGHV3-43*02                 AGCTCTGGGAGAGGAGCCCCAGCCCTGAGATTCCCAGGTGTTTCCATTCGGTGATCAGCACTGAACACAGAGAACTCACCATGGAGTTTGGACTGAGCTGGGTTTTCCTTGTTGCTATTTTAAAAGGTGTCCAGTGT
IGHV3-20*01/*04             AGCTCTGGGAGAGGAGCCCCAGCCCTGAGATTCCCACGTGTTTCCATTCAGTGATCAGCACTGAACACAGAGGACTCGCCATGGAGTTTGGGCTGAGCTGGGTTTTCCTTGTTGCTATTTTAAAAGGTGTCCAGTGT
IGHV3-9*01/*03              AGCTCTGGGAGAGGAGCCCCAGCCCTGAGATTCCCAGGTGTTTCCATTCAGTGATCAGCACTGAACACAGAGGACTCACCATGGAGTTGGGACTGAGCTGGATTTTCCTTTTGGCTATTTTAAAAGGTGTCCAGTGT
```

Supplementary Figure 2. Upstream region sequences of IGHV3-43D, IGHV3-43 and IGHV3-9, which in parts are very similar.

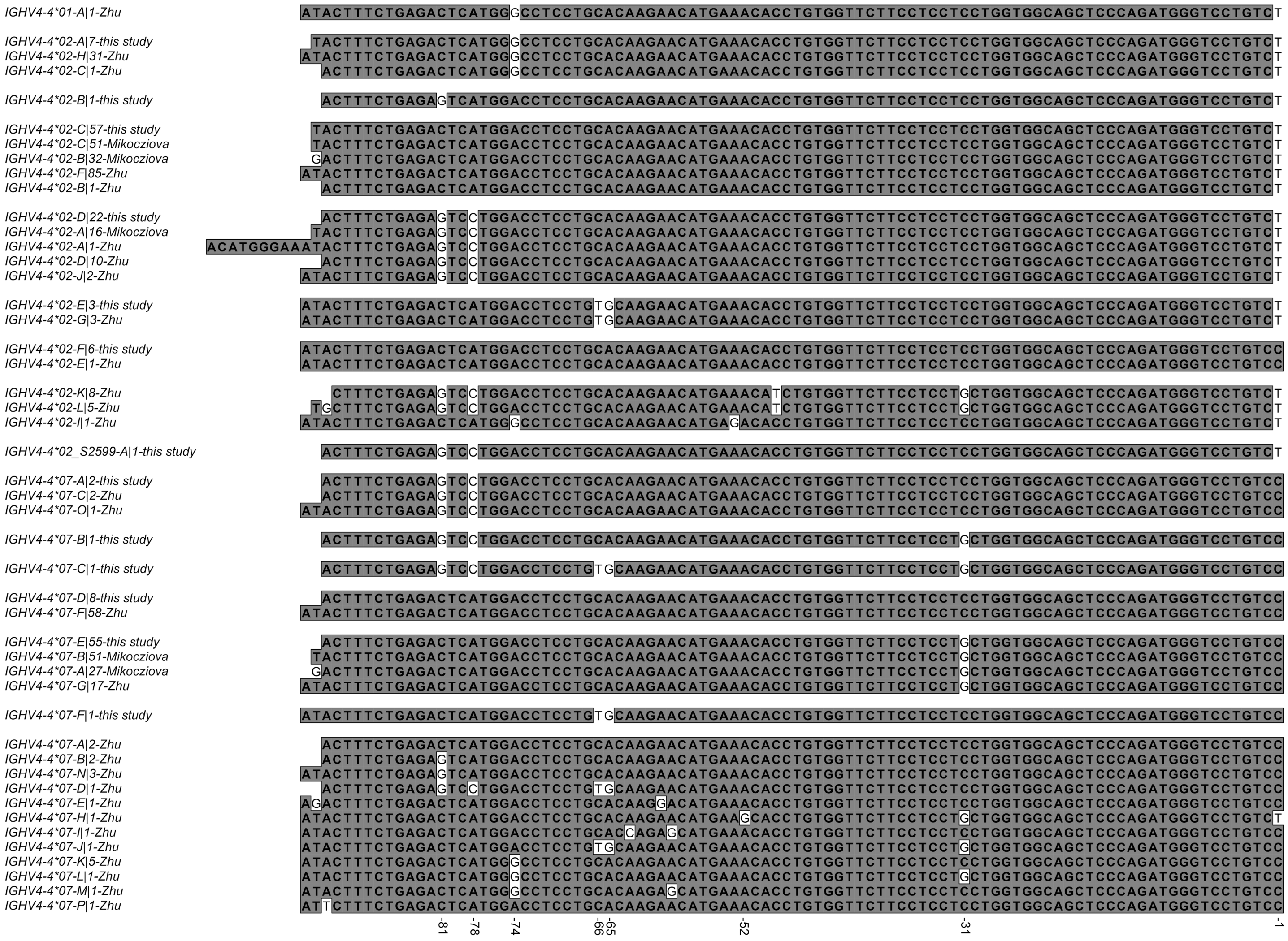

Supplementary Figure 3. The alignment of inferred upstream regions of alleles IGHV4-4*01, IGHV4-4*02 and IGHV4-4*07 as described in this study, Mikocziova et al. (18), and Zhu et al. (14). The sequences from Mikocziova et al.'s study and this study were inferred from exactly the same data set as used in this study. Names of upstream regions indicate "the IGHV allele to which the sequence was found to be associated – a letter separating different sequences associated to this allele number of instances in which it was found – study of identification".

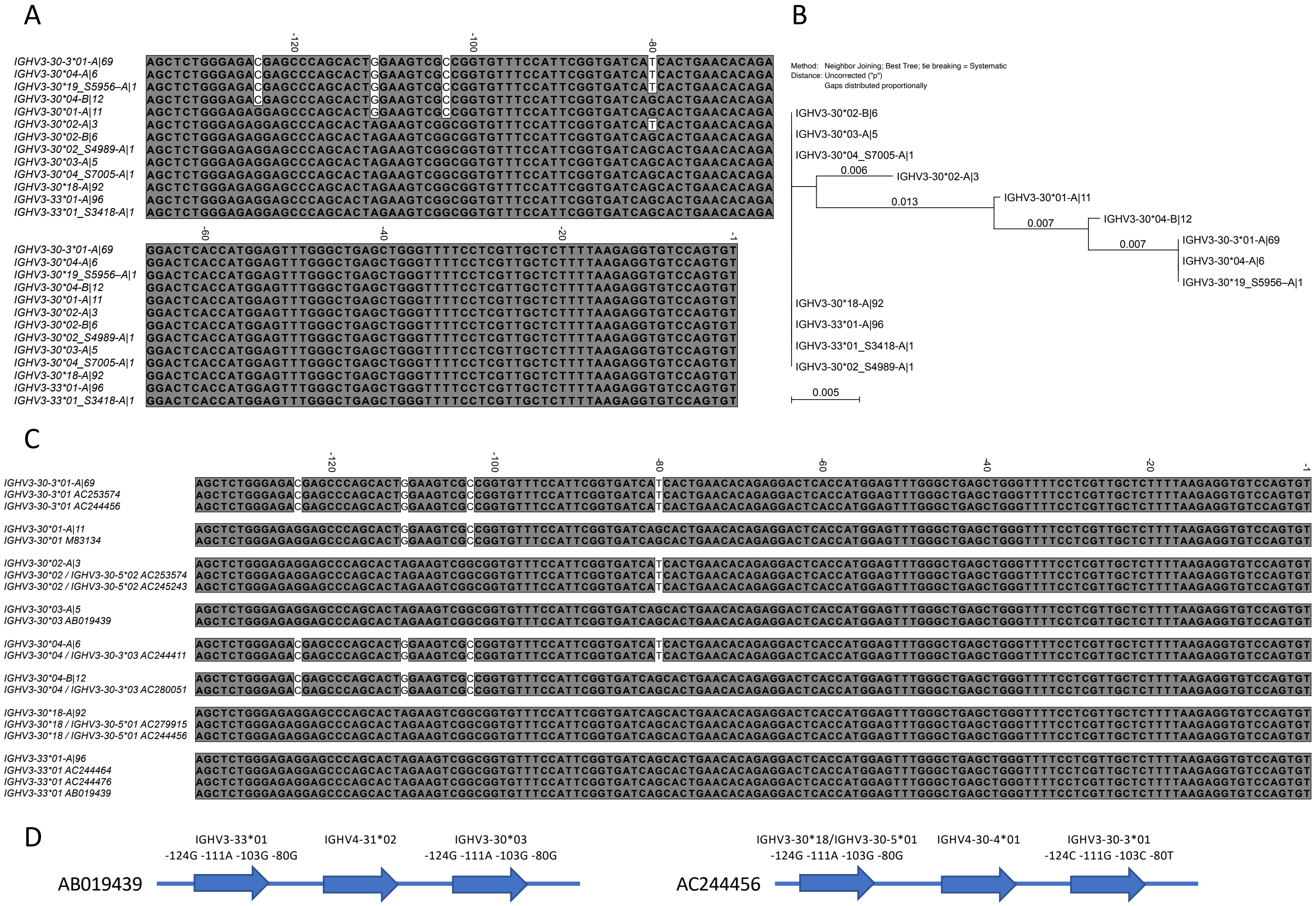

Supplementary Figure 4. Sequences (**A**) and Neighbour Joining Tree (**B**) of 5'UTR-leader sequences of alleles of IGHV3-30, IGHV3-30-3, and IGHV3-33. Examples of 5'UTR-leader sequences identified by inference have also been seen in genomic sequence data (genomic sequences are shown after removal of the leader intron; accession numbers are shown for selected cases) (**C**). Examples of part of the IGHV locus that also include alleles of IGHV3-30, IGHV3-30-3, IGHV3-30-5, and IGHV3-33 are illustrated (**D**).

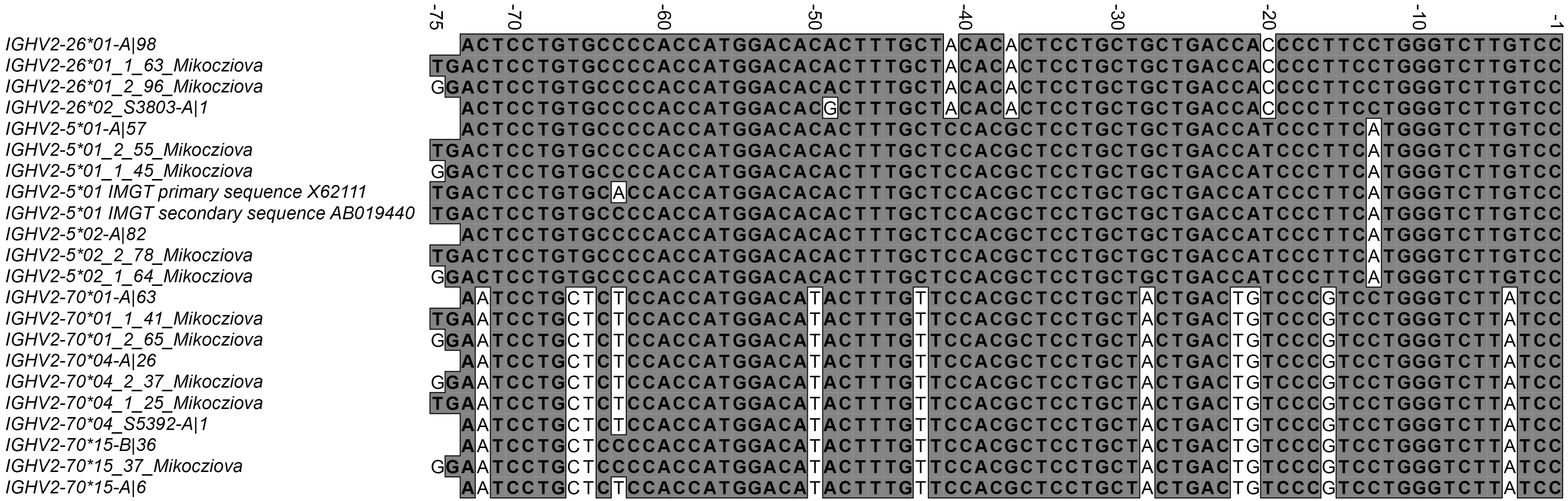

Supplementary Figure 5. Sequences of inferred upstream regions of genes/alleles of the IGHV2 subgroup (this study and Mikocziova et al [18]), and of the upstream regions of IGHV2-5*01 represented in two entries of the IMGT database, one of which features a C->A variant in base -63. This variant is not seen in human populations (highest population MAF<0.01%). The variant (T->G) previously inferred at high frequency in position -75 (18) is not reproduced in population studies as this base is represented by T at a frequency of 99.99% in IGHV2-26, IGHV2-5, and IGHV2-70 in studied human populations (http://www.ensembl.org) (28).

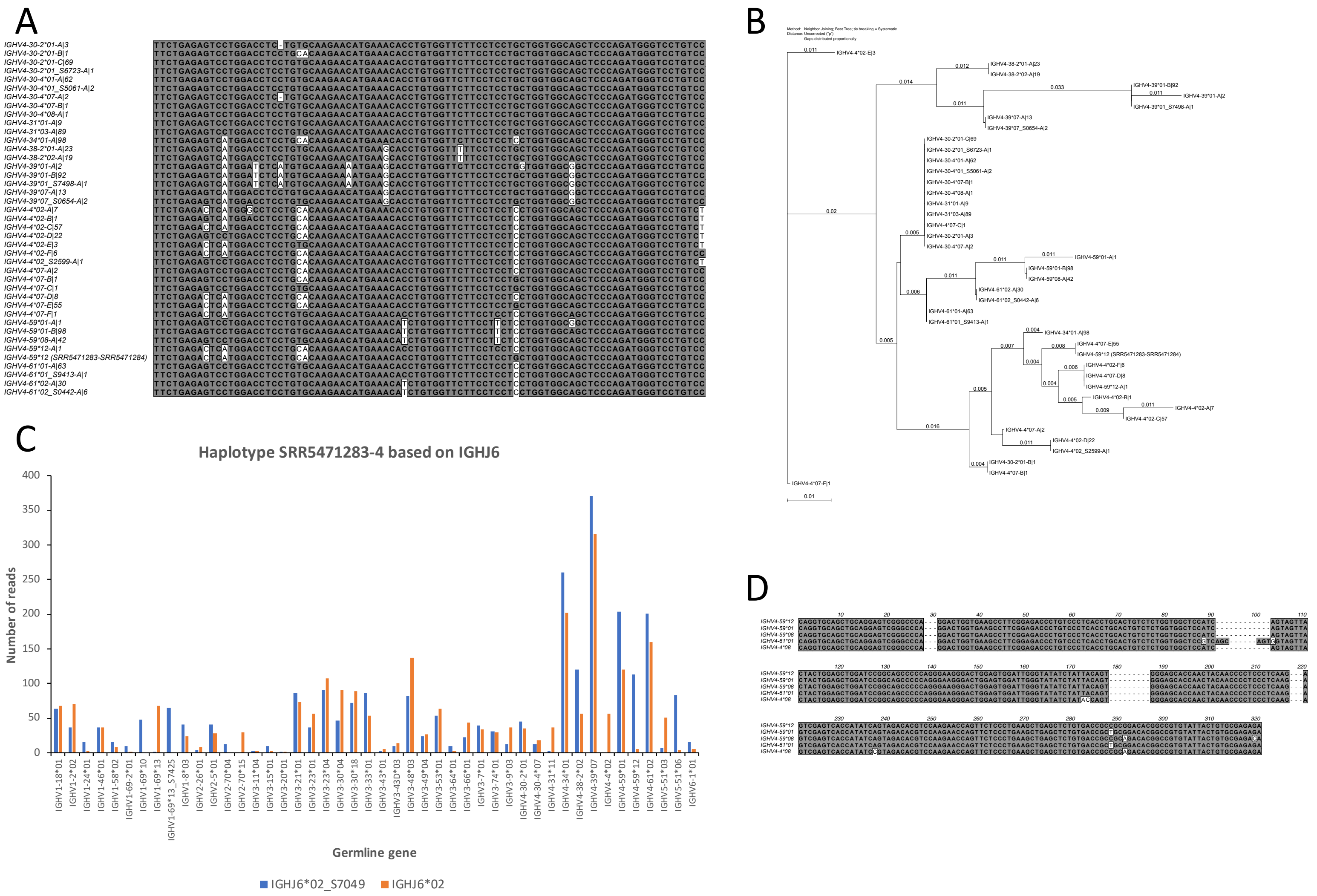

Supplementary Figure 6. Sequences (**A**) and neighbour Joining Tree (**B**) of upstream regions of genes belonging to subgroup 4, and haplotype analysis (**C**) of the individual (donor LP08248) where IGHV4-59*12 originally was identified (24). Upstream regions of IGHV4-59*12 are more closely related to upstream regions of most alleles of IGHV4-4 than to other upstream regions of alleles of IGHV4-59. Similarity of some alleles of IGHV4-4, IGHV4-59, and IGHV4-61 is shown (**D**).

A

Common inference allele and upstream region calls of IGHV1-69 and IGHV2-70 in haplotypes of 35 subjects in which haplotyping based on heterozygocity of IGHJ6 could be performed.

Inferred allele of IGHV1-69/ IGHV1-69D		Inferred allele of IGHV1-69-2	Inferred allele of IGHV2-70		Total number out of 70 haplotypes
Allele	Upstream sequence		Allele	Upstream sequence	
IGHV1-69(D)*01 (n=14)<br>IGHV1-69*09 (n=1)	-88A<br>-100C	None	IGHV2-70*01	-63T	15
IGHV1-69*02 (n=18)<br>IGHV1-69*04 (n=8)<br>IGHV1-69*10 (n=2)<br>IGHV1-69*12 (n=2)	-88A<br>-100G	None	IGHV2-70*15<br>(n=16)<br>or<br>None†<br>(n=14)	-63C	30
IGHV1-69(D)*01 (n=15)<br>IGHV1-69*06 (n=1)	-88A<br>-100C	IGHV1-69-2*01<br>(n=15)<br><br>None (n=1)	IGHV2-70*01 &<br>IGHV2-70(D)*04	-63T &<br>-63T	16
IGHV1-69*06 (n=13)<br>IGHV1-69*09 (n=2)<br>IGHV1-69*17 (n=1)	-88G<br>-100C				
IGHV1-69(D)*01	-88A<br>-100C	IGHV1-69-2*01<br>(n=15)	IGHV2-70(D)*04 &<br>IGHV2-70*15	-63T &<br>-63T	3
IGHV1-69*06	-88G<br>-100C				

† In some samples reads of alleles of IGHV2-70 could be seen, but their diversity and numbers were insufficient to permit inference using standard inference tool settings. In some subjects reads of what appears as representing novel alleles of IGHV2-70 could be seen.

B

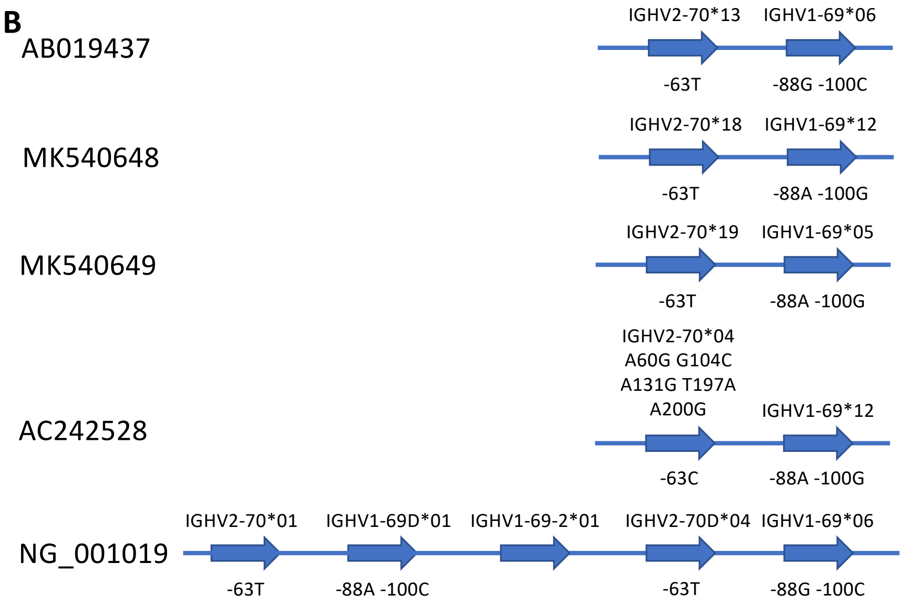

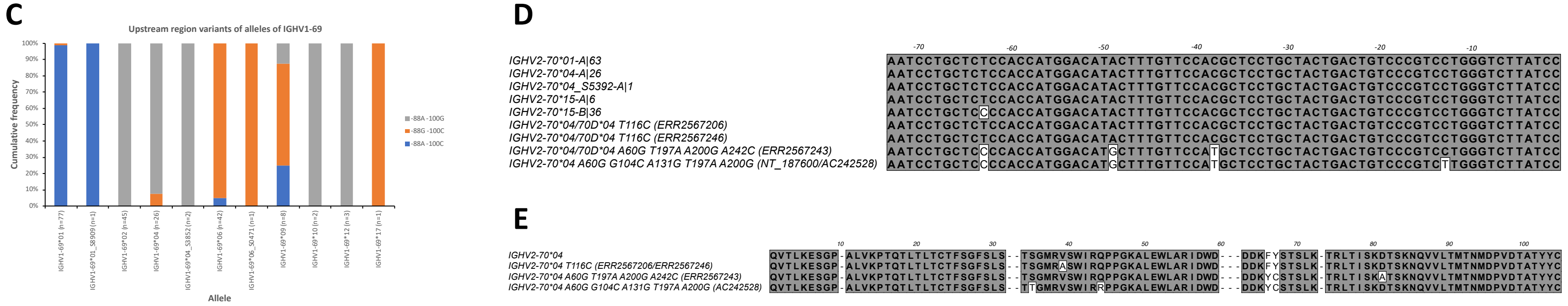

Supplementary Figure 7. Common variants of the IGHV1-69 and IGHV2-70 region of human haplotypes, including duplicated variants of this region, as represented by alleles of the genes and variants of their upstream regions identified by inference technology (**A**). Examples of germline this part of the IGHV locus as defined by genomic sequencing (**B**). Different alleles of the IGHV1-69/IGHV1-69D genes are primarily associated to different upstream region sequence variants (**C**). Commonly inferred alleles of IGHV2-70 are commonly associated to the same upstream region, although some instances of IGHV2-70*15 are associated to a different upstream region (**D**), in which case it also occurs in a different genomic context (**A**). In addition, in the transcriptome of some individuals, rare reads of additional variants of IGHV2-70 were identified (**E**), alleles that had not been inferred, one of which also encoded unusual residues including a cysteine in framework 3. One such allele has also been identified by genomic sequencing (**B, E**). Some of these alleles were associated to upstream regions different from those of commonly expressed alleles of IGHV2-70 (**D**). IGHV2-70*04/70D*04 T116C represents a 5' and 3'-extended version of IGHV2-70*05, an allele that is currently only partly defined in the IMGT database (GenBank accession number: Z27502).

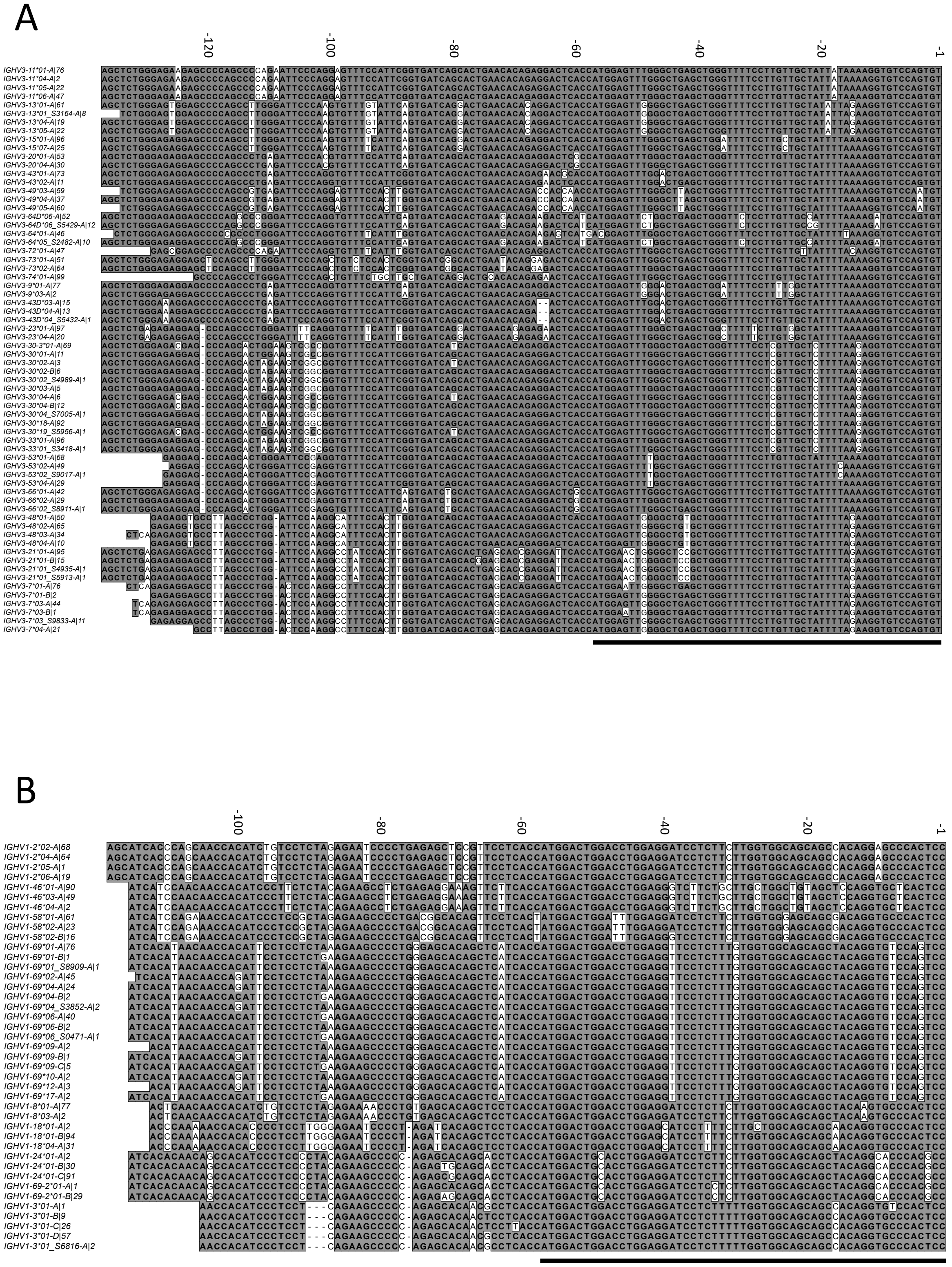

Supplementary Figure 8. Inferred upstream regions of genes/alleles of IGHV3 (**A**) and IGHV1 (**B**), grouped according to the presence of possible indels in the 5'UTR. The signal-peptide-encoding leader sequences are indicated by a solid line.

A

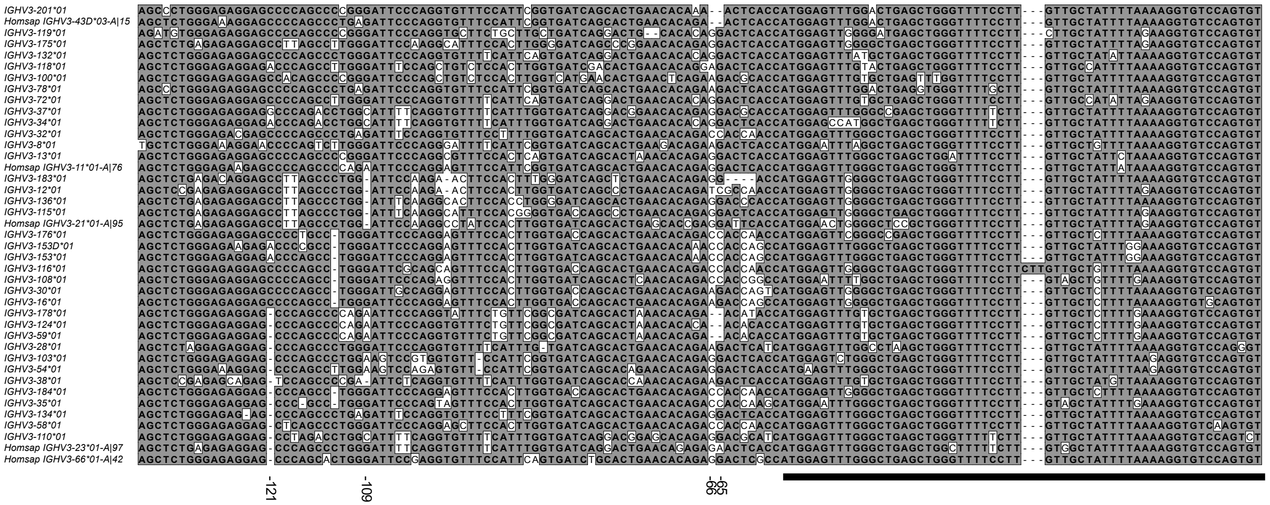

B

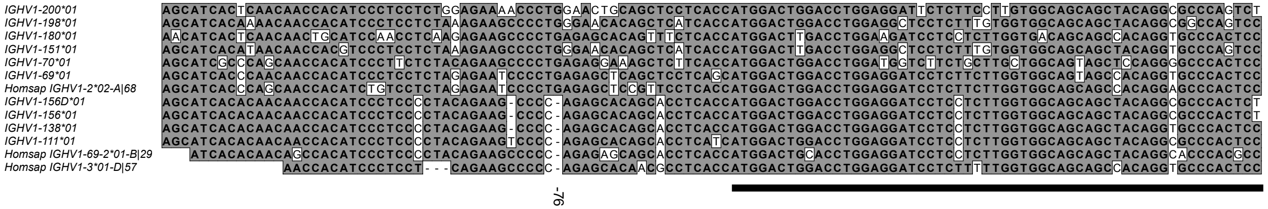

Supplementary Figure 9. Sequences of upstream regions (5'UTR-leader sequences excluding the intron found in the leader sequence) of functional genes of Rhesus monkey subgroups IGHV3 (**A**) and IGHV1 (**B**) as defined by IMGT entry IMGT000064 (http://www.imgt.org/ligmdb/view?id=IMGT000064). Examples of the corresponding inferred upstream regions of human alleles (all with the prefix "Homsap") representing sequences of different lengths or showing evidence of insertion/deletion events are included. The sequences encoding the gene products' signal sequences are underlined.

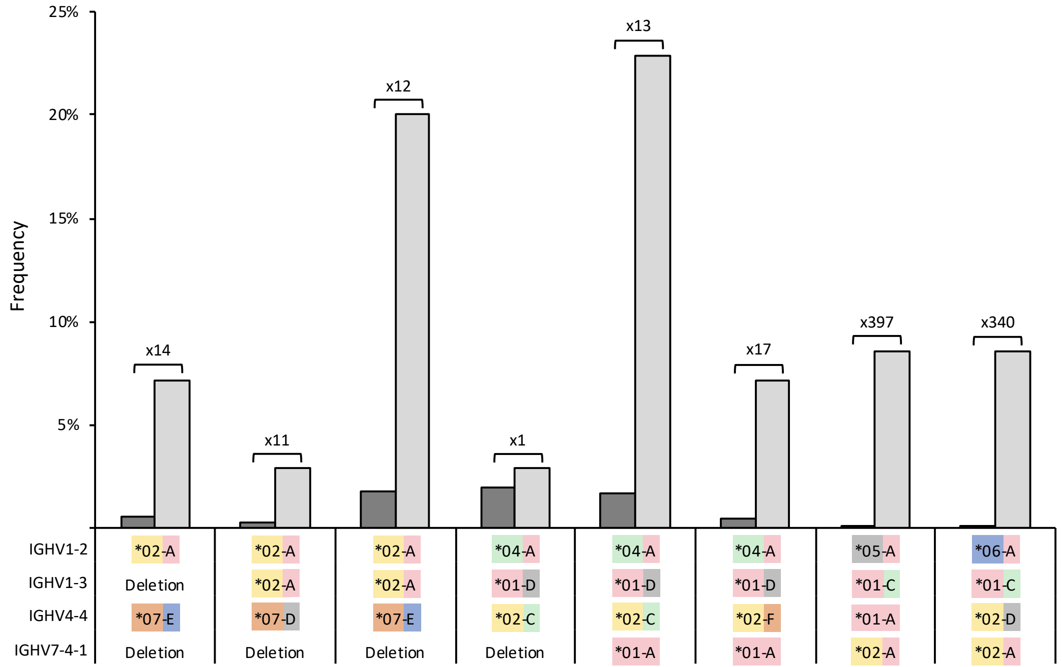

Supplementary Figure 10. Frequency of different gene-allele-upstream region combinations (light grey) in the 35 haplotypable individuals in the studied data set, together with theoretical frequencies (dark grey) (assuming random association of alleles). Colors below x-axis indicate allele of each gene (*01 – pink, *02 – yellow, *04 – green, *05 – grey, *06 – blue, and *07 – brown) and the upstream region sequence (A – pink, C – green, D – grey, E – blue, and F – brown). Values above bars show the ratio between the theoretical and actual frequency in the given 70 haplotypes. Gene combinations present in <2 haplotypes are not shown.
